# Efficient Photosynthetic Functioning of *Arabidopsis thaliana* Through Electron Dissipation in Chloroplasts and Electron Export to Mitochondria Under Ammonium Nutrition

**DOI:** 10.3389/fpls.2020.00103

**Published:** 2020-02-26

**Authors:** Anna Podgórska, Radosław Mazur, Monika Ostaszewska-Bugajska, Katsiaryna Kryzheuskaya, Kacper Dziewit, Klaudia Borysiuk, Agata Wdowiak, Maria Burian, Allan G. Rasmusson, Bożena Szal

**Affiliations:** ^1^ Institute of Experimental Plant Biology and Biotechnology, Faculty of Biology, University of Warsaw, Warsaw, Poland; ^2^ Institute of Biochemistry, Faculty of Biology, University of Warsaw, Warsaw, Poland; ^3^ Department of Biology, Lund University, Lund, Sweden

**Keywords:** nitrogen assimilation, ammonium toxicity syndrome, photosynthetic efficiency, redox dissipation, alternative oxidase, oxidative damage, non-photochemical quenching, redox export

## Abstract

An improvement in photosynthetic rate promotes the growth of crop plants. The sink-regulation of photosynthesis is crucial in optimizing nitrogen fixation and integrating it with carbon balance. Studies on these processes are essential in understanding growth inhibition in plants with ammonium (${\mathrm{NH}}_{4}^{+}$) syndrome. Hence, we sought to investigate the effects of using nitrogen sources with different states of reduction (during assimilation of ${\mathrm{NO}}_{3}^{-}$ versus ${\mathrm{NH}}_{4}^{+}$) on the photosynthetic performance of *Arabidopsis thaliana*. Our results demonstrated that photosynthetic functioning during long-term ${\mathrm{NH}}_{4}^{+}$ nutrition was not disturbed and that no indication of photoinhibition of PSII was detected, revealing the robustness of the photosynthetic apparatus during stressful conditions. Based on our findings, we propose multiple strategies to sustain photosynthetic activity during limited reductant utilization for ${\mathrm{NH}}_{4}^{+}$ assimilation. One mechanism to prevent chloroplast electron transport chain overreduction during ${\mathrm{NH}}_{4}^{+}$ nutrition is for cyclic electron flow together with plastid terminal oxidase activity. Moreover, redox state in chloroplasts was optimized by a dedicated type II NAD(P)H dehydrogenase. In order to reduce the amount of energy that reaches the photosynthetic reaction centers and to facilitate photosynthetic protection during ${\mathrm{NH}}_{4}^{+}$ nutrition, non-photochemical quenching (NPQ) and ample xanthophyll cycle pigments efficiently dissipate excess excitation. Additionally, high redox load may be dissipated in other metabolic reactions outside of chloroplasts due to the direct export of nucleotides through the malate/oxaloacetate valve. Mitochondrial alternative pathways can downstream support the overreduction of chloroplasts. This mechanism correlated with the improved growth of *A. thaliana* with the overexpression of the alternative oxidase 1a (AOX1a) during ${\mathrm{NH}}_{4}^{+}$ nutrition. Most remarkably, our findings demonstrated the capacity of chloroplasts to tolerate ${\mathrm{NH}}_{4}^{+}$ syndrome instead of providing redox poise to the cells.

## Introduction

Photosynthesis enables several anabolic reactions and provides energy for the metabolism and growth of plants. Unfavorable changes in the environment are quickly perceived by plants to minimize disturbances in electron transport flows. Different stress factors, such as changes in light intensity or nutrient availability, affect the intracellular metabolism of plants, and consequently, fluctuations in energy or redox state develop in cells. One natural factor, which directly affects chloroplast redox state, is the use of different nitrogen sources. Electrons (é) from the photosynthetic electron transport chain (chlETC) can be used for nitrogen assimilation into amino acids. The bioenergetically expensive version involves the nourishment of plants with nitrate (${\mathrm{NO}}_{3}^{-}$), which initially undergoes a two-step reduction. In this case, ${\mathrm{NO}}_{3}^{-}$ reduction is expected to consume up to 20% of the total reductants produced by photosynthetic light reactions (Noctor and Foyer, 1998) because of the activity of nitrite reductase (NiR) accepting 6é and of 2-oxoglutarate aminotransferase (GOGAT) accepting 2é. The efficiency of their function is dependent on the transfer of electrons directly from reduced ferredoxin (Fd), and consequently they compete with ferredoxin NADP^+^ oxidoreductase (FNR), thereby decreasing the nicotinamide adenine dinucleotide phosphate (NADPH) formation. The alternative to ${\mathrm{NO}}_{3}^{-}$ assimilation is the instant growth of plants on ammonium (${\mathrm{NH}}_{4}^{+}$), which does not need to be reduced, thereby saving much of the cell's reducing power. Surprisingly, when plants are nourished with ${\mathrm{NH}}_{4}^{+}$ as the sole source of nitrogen, developmental disorders arise (Britto and Kronzucker, 2002; Bittsánszky et al., 2015; Esteban et al., 2016). Most crop plants show strong growth inhibition and other toxicity symptoms (Podgórska and Szal, 2015; Liu and von Wirén, 2017) in response to ${\mathrm{NH}}_{4}^{+}$ nutrition, making this condition largely considered to be a severe one. However, the cause of this syndrome is yet to be elucidated. It was previously hypothesized that ${\mathrm{NH}}_{4}^{+}$ could primarily act as a photophosphorylation uncoupler and could consequently disrupt photosynthesis, but this was negated, considering that free ${\mathrm{NH}}_{4}^{+}$ could not accumulate to dangerously high concentrations in plant tissues (Gerendás et al., 1997; Zhu et al., 2000). After ${\mathrm{NH}}_{4}^{+}$ ions are taken up, they are quickly incorporated into amino acids through continuous GOGAT activity to prevent poise of the cell. It was further hypothesized that during stress conditions, that are causing the overreduction of chloroplasts, including ${\mathrm{NH}}_{4}^{+}$ nutrition, this could consequently lead to photoinhibition (Zhu et al., 2000; Guo et al., 2005). Moreover, when NADPH is accumulating and not enough terminal acceptor sides (NADP^+^) are available for the chlETC electron flow, the receiving molecule would be molecular oxygen (O_2_) in the Mehler reaction that results in the formation of reactive oxygen species (ROS). In our previous study, we have demonstrated that in contrast to whole tissues or plant mitochondria, in chloroplasts neither overreduction nor signs of oxidative stress were detected during the long-term growth of *Arabidopsis thaliana* with ${\mathrm{NH}}_{4}^{+}$ (Podgórska et al., 2013). Yet how chloroplasts can deal with the rigorous changes in redox homeostasis during ${\mathrm{NH}}_{4}^{+}$ nutrition has remained unknown.

The safe function of photosynthetic light reactions is based on the synchronized activity of photosystem I (PSI) and photosystem II (PSII) in the linear photosynthetic electron transfer (LET) chain. Hence, efficient electron sinks for PSI are highly desired to minimize the risk of photoinhibitory conditions and ROS generation when ${\mathrm{NH}}_{4}^{+}$ is supplied. The Mehler reaction can also be considered as electron drainage, considering that one electron is reconstituted for superoxide anion radical (O_2_˙^-^) generation. In order to promptly minimize the damage, superoxide dismutase (SOD) reduces O_2_˙^-^ to hydrogen peroxide (H_2_O_2_), which is scavenged in an ascorbate-dependent reaction. Hence, potent antioxidant systems are necessary to reduce ROS accumulation in chloroplasts. Plants also have other mechanisms to prevent photodamage, and they accomplish so by utilizing excess reductants when the photon flux density exceeds the energy requirement of CO_2_ fixation, nitrogen assimilation, and other anabolic reactions. One of the mechanisms that balance reductants and energy demand is cyclic electron transport (CET), which functions in the chlETC without the accumulation of NADPH (Munekage et al., 2002). This cycle is branched into two independent pathways, including the NADH dehydrogenase-like (NDH) complex constitutively associating with PSI and the route mediated by proton gradient regulation 5 (PGR5) and PGR5-like photosynthetic phenotype 1 (PGRL1) proteins (Suorsa et al., 2016). Furthermore, the plastid terminal oxidase (PTOX), can receive electrons from the plastoquinol (PQH_2_) pool. Another way to prevent redox input at PSI is to limit the electron flow from PSII. Also damage to PSII is a valid risk. Excess light energy absorbed by the antenna proteins of PSII can be dissipated as heat through the non-photochemical quenching (NPQ) process, which consists of four components. The major component of NPQ is qE, which involves the dissipation of excess light energy in the antenna light-harvesting complex II (LHCII) to heat before it reaches the reaction center. Another component is the state transition (qT), which occurs when light adsorption is balanced between the photosystems through the relocation of LHCII proteins in proximity to PSI. Another important component of NPQ is the temporary photoinhibition of PSII (qI), an effect that can be induced by the damage of D1 protein in PSII. Another component is the zeaxanthin dependent quenching (qZ), in which xanthophyll cycle carotenoids trigger NPQ after the conversion of violaxanthin to zeaxanthin in the process of de-epoxidation.

The main metabolic pathway influencing the redox and energy state of chloroplasts is the Calvin-Benson-Bassham (CBB) cycle, which starts with the carboxylation activity of ribulose-1,5-bisphosphate-carboxylase/oxygenase (RUBISCO). The regulation of the redox status of the chloroplasts is also accomplished through photorespiration, a process initiated by the oxygenase activity of RUBISCO. In the photorespiratory metabolic pathway, the reactions localized in the chloroplasts, peroxisomes, and mitochondria are engaged. Moreover, a direct way to regulate the chloroplastic redox homeostasis is the redox-shuttling activity of the oxaloacetate/malate (OAA/Mal) valve (Selinski and Scheibe, 2014). The reduction of oxaloacetate to malate by the NADP^+^-dependent malate dehydrogenase (NADP^+^-MDH) gives rise to malate export from chloroplast. Subsequently, malate is then transported from the cytosol to the mitochondrial matrix. Both the processes of photorespiration and the OAA/Mal valve can be seen as the transfer of reducing power from chloroplasts to mitochondria. In these processes, mitochondrial NADH is produced by the activity of glycine carboxylase complex (GDC) or mitochondrial NAD^+^-MDH respectively.

Mitochondria are essential as downstream electron acceptors during NH_4_⁺ nutrition (Guo et al., 2005). External or internal type II dehydrogenases can directly accept electrons, alternatively to complex I (NADH dehydrogenase), in the mitochondrial electron transport chain (mtETC), but this process cannot be completed without the alternative oxidase (AOX), which has the unique function of transporting electrons from reduced ubiquinone to molecular oxygen (Vanlerberghe, 2013). Clearly, these alternative pathways are important in preventing overreduction in chloroplasts and in balancing whole-cell redox state (Vishwakarma et al., 2015). In transgenic plants, there is strong evidence that the major role of AOX is to prevent ROS production in the mtETC (Cvetkovska and Vanlerberghe, 2012). The most abundant AOX gene in *Arabidopsis* is *AOX1a,* and it responds to several stresses (Clifton et al., 2006).

Although it has been widely accepted that nitrogen assimilation is closely linked with photosynthetic performance, their primary effects on plant functioning are yet to be characterized. Moreover, the effects of different nitrogen growth regimes, differing in their reduction state whether ${\mathrm{NO}}_{3}^{-}$ or ${\mathrm{NH}}_{4}^{+}$ is used as a nutrient, can affect these processes. In this study, we aimed to determine the major protecting machinery of the photosynthetic apparatus of plants challenged with ${\mathrm{NH}}_{4}^{+}$ nutrition. This knowledge is vital in understanding the fine-tuning of photosynthesis and the resistance of plants to environmental stresses.

## Materials and Methods

### Plant Samples and Growth Conditions

Experiments were performed on *A. thaliana* ecotype Columbia-0 wild type (WT) and on derived transgenic plants, which were transformed with *AOX1a* under the control of the CaMV 35S promoter selected by Umbach et al. (2005). The incorporation of sense-constructs gave rise to the overexpressor (XX-2) mutants, while the insertion of the antisense-construct created the suppressor (AS-12) line. After their seed germination in 1% agar in ½ x Murashige & Skoog for one week, the plants were supplemented with nutrient medium (Lasa et al., 2002). The hydroponics cultures in an Araponics growth system (Liege, Belgium) were established for 8 weeks, while the liquid medium was renewed twice a week. The plants were differentiated into receiving either of the two exclusive sources of nitrogen: 5 mM ${\mathrm{NO}}_{3}^{-}$ or 5 mM ${\mathrm{NH}}_{4}^{+}$, as previously done by Podgórska et al. (2013). The culture was maintained for 8 h in 150 µmol m^-2^ s^-1^ photosynthetically active radiation (PAR; daylight and warm white 1:1, LF-40W, Piła, Poland) at 21 °C and for 18 h at dark at 18 °C, during the day/night cycle. The WT plants nourished with ${\mathrm{NO}}_{3}^{-}$ were considered as the control. All the assays were carried out on leaf samples or organelles isolated at 12:00, after 3 h of illumination.

To analyze their phenotypic distinction, several rosettes were randomly harvested from independent plant cultures grown with either ${\mathrm{NO}}_{3}^{-}$ or ${\mathrm{NH}}_{4}^{+}$. Subsequently, their respective fresh weight (FW) was determined.

### Parameters of Photosynthetic Performance

*In vivo* gas exchange parameters on well-developed leaves were determined using an infrared gas analyzer (LI-6800; LI-COR, Lincoln, NE, USA). The photosynthetic rate of each leaf was measured at atmospheric CO_2_ concentration of 400 µmol CO_2_ mol^-1^ air at 22 °C and under the light intensity of 150 µmol photons m^-2^ s^-1^ at 90% red light with 70% humidity. The net assimilation rate (A_net_) of CO_2_ was calculated per leaf area.

Chlorophyll (chl) *a* fluorescence images were recorded using the Maxi version of Imaging-PAM chlorophyll fluorescence system (Heinz Walz, Germany). Before they were measured, the *A. thaliana* rosettes were dark-adapted for at least 30 min. The fluorescence images were recorded with a resolution of 640 × 480 pixels and with the camera parameters set to avoid saturation of the charge-coupled device (CCD) wells. Minimal (F_0_) fluorescence was determined using weak blue modulating light of 0.5 μmol photons m^−2^ s^−1^, whereas maximal (F_M_) fluorescence was measured through 0.84 s of saturation blue light pulse with 2,700 μmol photons m^−2^ s^−1^. After 60 s of dark relaxation the blue actinic light of 391 μmol photons m^−2^ s^−1^ was employed, and saturation pulses at 20-s time intervals were applied. After 200 s, actinic light was switched off, and additional saturation pulses for approximately 15 min of dark relaxation were applied. At all saturation pulses, the maximum (F_M_') fluorescence values were measured, and the minimum (F_0_') fluorescence was calculated as F_0_' = F_0_(F_V_/F_M_ + F_0_/F_M_′). The recorded data were analyzed using ImagingWinGigE software. The photosynthetic parameters: F_V_/F_M_, Y(II), NPQ, and qL were calculated according to the formulae previously specified by Mazur et al. (2016). The qE and qI components of NPQ ware calculated according to Müller et al. (2001).

### Isolation of Chloroplasts and Thylakoids

Intact chloroplasts were isolated, according to the method of Romanowska and Albertsson (1994) with minor modifications. Each fresh leaf tissue weighing 2 g was homogenized using a knife blender in 40-mL cool preparation medium composed of 4-(2-hydroxyethyl)-1-piperazineethanesulfonic acid HEPES-KOH with pH 7.4, 330 mM mannitol, 1 mM MgCl_2_, 5 mM NaCl, 1 mM ethylenediaminetetraacetic acid (EDTA), and 10 mM NaF. After its filtration through an 80-μm nylon mesh, each homogenate was centrifuged at 3,600 × g for 6 min. The pellet was resuspended in suspension buffer containing 20 mM HEPES-KOH with pH 7.4, 330 mM sorbitol, 1 mM MgCl_2,_ 5 mM NaCl, and 10 mM NaF and was washed twice using the same washing buffer. The isolated chloroplasts were ground in potter homogenizer.

Thylakoid membranes were isolated according to Garstka et al. (2005) as modified in Garstka et al. (2007). Each fresh leaf tissue weighing 2 g was homogenized using a knife blender in 40-ml cool preparation medium composed of 20 mM Tricine-NaOH with pH 7.5, 330 mM sorbitol, 40 mM L-ascorbic acid, 15 mM NaCl, 4 mM MgCl_2_, and 10 mM NaF. After its filtration through an 80-μm nylon mesh, the homogenate was centrifuged for 4 min at 2,000 × g. The pellet was agitated in a buffered medium (20 mM Tricine–NaOH with pH 7.0, 15 mM NaCl, and 4 mM MgCl_2_) and subsequent centrifugation at 6,000 × g for 10 min was performed. The obtained thylakoid membranes were washed twice in suspension buffer.

### Evaluation of Photochemical Efficiency

The electron flow between both photosystems was assayed as light dependent oxygen evolution in the Clark electrode (Hansatech). The maximum photochemical capacity of PSII and PSI was measured in isolated chloroplasts using 0.1 mM methylviologen (MV) as an electron acceptor and with 5 mM sodium azide.

The corresponding maximal capacities of PSI and PSII were spectrophotometrically measured, as described by Vernon and Cardon (1982). The total activity of PSI in light was determined as the oxidation of 0.2 mM N,N,N′,N′-tetramethyl-*p*-phenylenediamine (TMPD) with 0.5 mM MV as the electron acceptor, whereas the total activity of PSII was measured in isolated chloroplasts as the direct reduction of 0.1 mM 2,6-dichlorophenolindophenol (DCPIP).

### Blue-Native PAGE and Two-Dimensional SDS PAGE Assays

For the native separation of protein complexes, blue native polyacrylamide gel electrophoresis (BN-PAGE) was performed, according to the methods of Rogowski et al. (2019) with modifications by Mazur et al. (2019). First thylakoid membranes were suspended in 1x native PAGE buffer from the sample preparation kit (Invitrogen, Carlsbad, California, USA) and were solubilized with 1% n-dodecyl β-D-maltoside (DDM). After centrifugation at 18,000 x g for 15 min, 1% Coomassie brilliant blue G-250 was added to the supernatant. Samples containing 8.3 µg of chlorophyll were separated on 4%−16%-gradient acrylamide gel (Invitrogen) in 1x anode and 1x cathode dark blue buffer (Invitrogen). During electrophoresis, the cathode buffer was changed to light blue version. Finally, the destaining of gels was performed with 0.5 M 6-aminocaproic acid in 25 mM imidazole-HCl with pH 7.0, and the gels were immediately scanned. The protein complexes were identified, and the abundance of PSI-NDH was quantified based on the densitometry of bands using Image-Lab 5.2 software (Bio-Rad), after the correction for background.

The detached protein lanes from BN-PAGE were utilized for the separation in second dimension, as described by Mazur et al. (2019). After the denaturation of the protein complexes in 125 mM Tris-HCl with pH 6.8, 5 M urea, 10% glycerol, 5% sodium dodecyl sulfate (SDS), and 5% β-mercaptoethanol for 30 min at 65°C, the gel stripes were separated in 12% acrylamide gel containing 6 M urea and 0.1% SDS. The gels were washed, and the gel staining was conducted in 0.1% silver nitrate (Shevchenko et al., 1996). The gels were scanned, and the visual analysis of the composition and arrangement of the chlorophyll protein complexes was performed.

### Analysis of Protein Levels

In order to determine their protein abundance, the extracts were subjected to SDS-PAGE, as previously described in Podgórska et al. (2018). Briefly, the leaf tissues were homogenized with 3x extraction buffer. To keep the AOX and NDC proteins reduced, the samples were supplemented with 100 mM 1,4-dithiothreitol (DTT). For the measurement of protein abundance, these extracts were correspondingly used to estimate the levels of AOX1/2 (15 µl), PTOX (5 µl), ascorbate peroxidase (APX, 15 µl), glycine decarboxylase complex H subunit (GDC-H) (5 µl), serine hydroxymethyltransferase (SHMT, 2.5 µl), and hydroxypyruvate reductase (HPR, 5 µl) as well as RUBISCO (2 µl of 10x diluted extracts). Furthermore, 9 µg chl of the isolated chloroplast extracts was used to measure the abundance of NDC1, and another 9 µg chl of the isolated thylakoid extracts was utilized to evaluate the concentration of ATPase α-subunit. The Western blotting of the primary antibodies: anti-AOX diluted at 1:1000, anti-PTOX diluted at 1:4000, anti-APX diluted at 1:2000, anti-GDC-H diluted at 1:5000, anti-SHMT diluted at 1:5000, anti-HPR diluted at 1:200, anti-RUBISCO diluted at 1:5000, anti-NDC1 diluted at 1:5000, and antiATPase α-subunit diluted at 1:5000 (all from Agrisera, Vännäs, Sweden except HPR) was conducted overnight at 4°C. Then it was followed by the incubation of the anti-rabbit secondary antibody conjugated to horseradish peroxidase (HRP) (diluted at 1:25000; Bio-Rad) for 1 h at room temperature (RT). After their chemiluminescence identification, the corresponding stained polypeptides were identified based on their molecular masses. The protein levels were quantified based on the densitometry of bands using Image-Lab 5.2 software (Bio-Rad), after the correction for background.

The levels of carbonylated protein derivatives in the isolated chloroplasts were quantified, according to the methods of Juszczuk et al. (2008) with minor modifications. After derivatization using 10 mM 2,4-dinitrophenylhydrazine (DNPH) in 10% trifluoracetic acid, the extracts containing 0.55 µg of chlorophyll were separated in 10% SDS-PAGE. Antibodies to the dinitrophenyl group (diluted at 1:1000; Sigma) were used as the primary antibodies, after which the anti-rabbit secondary antibodies (diluted 1:25000; Bio-Rad) were utilized. The amounts of oxidized proteins were visualized through chemiluminescence, and the staining intensity of the entire blot lane was quantified through densitometry using Image-Lab 5.2 software (Bio-Rad), after the correction for background.

### Measurement of Enzyme Activity and Protein Content

The SOD isoforms in the isolated chloroplasts were identified in 12% native acrylamide gel, as described by Sehmer et al. (1998). The exposure of bright areas was based on the inhibition of nitroblue tetrazolium (NBT) reduction by SOD activity and was quantified through the densitometry of bands using Image-Lab 5.2 software (Bio-Rad), after the correction for background.

The activity of NAD^+^-MDH was detected after the separation of foliar protein extracts in 8% native acrylamide gel, as described by Beeler et al. (2014). The enzyme activity was developed with 1 mM malate, 0.5 mM NAD^+^, 0.4 mM NBT, 27 µM phenazine methosulfate, and 15 mM MgCl_2_ in 100 mM Tris-HCl with pH 8.0. The exposure of purple formazan bands was quantified through the densitometry of bands using Image-Lab 5.2 software (Bio-Rad), after the correction for background. The activity of NADP^+^-MDH was spectrophotometrically assayed as the NADPH-dependent reduction of OAA (Keryer et al., 2004). The maximal enzyme activity was measured after the reductive activation of extracts with 10 mM DTT for 30 min at RT.

Protein concentration was measured according to the methods of Bradford (1976) using bovine serum albumin as the standard.

### Detection of Oxidative Stress-Related Metabolites

The H_2_O_2_ content of the leaf tissues was quantified based on the peroxidase-coupled assay, as described by Veljovic-Jovanovic et al. (2001). The accuracy of each reaction was controlled through internal standard determination, respectively applying 5 mM H_2_O_2_ to the same reaction mixture.

The low-mass antioxidants of leaf tissues were also measured. Ascorbic acid concentration was determined through the colorimetric bi-pirydyl method described by Okamura (1980). The levels of the reduced form of ascorbate (AsA) and dehydroascorbate (DHA) were calculated in the presence and absence of DTT.

The extent of lipid peroxidation in membranes was estimated by tracing malondialdehyde (MDA) concentration (Hodges et al., 1999). The content of MDA was corrected for interfering compounds in samples, where the specific substrate thiobarbituric acid was not added to the reaction mixture.

### Quantitative Real Time Polymerase Chain Reactions

RNA isolation, complementary DNA generation, and RNAse digestion were conducted, as described by Podgórska et al. (2018). Quantitative real-time polymerase chain reactions (RT-qPCR) were performed at an annealing temperature of 60 °C for all genes. Transcript abundance was normalized to the transcript level of the reference gene protein phosphatase 2A (*PP2A*), as described by Czechowski et al. (2005), and the results were expressed in relation to the WT plants grown on ${\mathrm{NO}}_{3}^{-}$ (value of 1), according to the method of Pfaffl (2001). New primer pairs were designed for *NADP-MDH* (5'–AGGGAGATGGAGATTATGAACTTG–3' and 5'–CAGTTCCGCTTCCGACTTG–3'), *PGR5* (5'–CCATTGCCTTACACTCTCAGGT–3' and 5'–AAGCCCTTGTCTCTGTTTTGC–3'), and NAD(P)H:plastoquinone oxidoreductase subunit L (*NDHL*) (5'–CCCAACGACACTCTTCTTCATAAT–3' and 5'–TGCTAAGGCTGGATGGTCAAT–3'). The *Arabidopsis* Genome Initiative locus identifiers for the genes investigated in this study were AT5G58330 (*NADP-MDH*), AT2G05620 (*PGR5*), AT1G70760 (*NDHL*), and AT1G13320 (protein phosphatase 2A, *PP2A*).

### Identification of Pigments

Chlorophyll was extracted in 80% acetone, and the concentration was calculated based on the extinction coefficients reported by Porra et al. (1989).

For the carotenoid analysis, pigments were extracted as described earlier (Szalonek et al., 2015). The extracted pigments were separated, according to the methods of Cuttriss et al. (2007) with some modifications, using Prominence HPLC system (Shimadzu). The 5-µl samples were injected into an Atlantis™ dC18 (3 µm, 100 Å, 3.0 x 150 mm) with Supelguard™ Ascentis™ C18 guard column (5 µm, 4.0 x 20 mm) and were eluted at 30°C with a constant flow rate of 1 ml min^-1^ using ethyl acetate gradient in acetonitrile, water, and triethylamine at 9:1:0.01 (v/v/v). The gradient was distributed with the following conditions: 0%−67% ethyl acetate in 1−31 min, 67%−100% ethyl acetate in 31−32 min, and 100% ethyl acetate with additional hold time of 1 min. In the next 2 min, the concentration of ethyl acetate was decreased to 0%, and the hold time was lengthened for 2 min more before the next injection. The separation of samples was monitored by the SPD-M20A prominence diode array detector (Shimadzu) at a range of 200−800 nm (1.2 nm resolution). The carotenoids were identified based on retention times and absorption spectra. For the quantification, the chromatogram at 436 nm was integrated using LCsolution v1.21 software (Shimadzu). The de-epoxidation state (DEPS) was calculated as (Z + 0.5A)/(Z + A + V), where Z, A, and V are zeaxanthin, antheraxanthin, and violaxanthin, respectively.

### Statistical Analysis

All data were expressed as the mean values ± standard deviations (SD) of 3–15 measurements taken from 3–5 independent plant cultures. The experimental data from different genotypes of the plants, which were nourished with either ${\mathrm{NO}}_{3}^{-}$ or ${\mathrm{NH}}_{4}^{+}$, were statistically analyzed through one-way analysis of variance (ANOVA) with Tukey's post-hoc test using Statistica 13.1 software (StatSoft, Inc., Tulsa, OK, USA). The results with p-value ≤ 0.05 were considered statistically significant.

## Results

### Oxidative Stress and Antioxidant Defense in Chloroplasts

**Figure 1:**
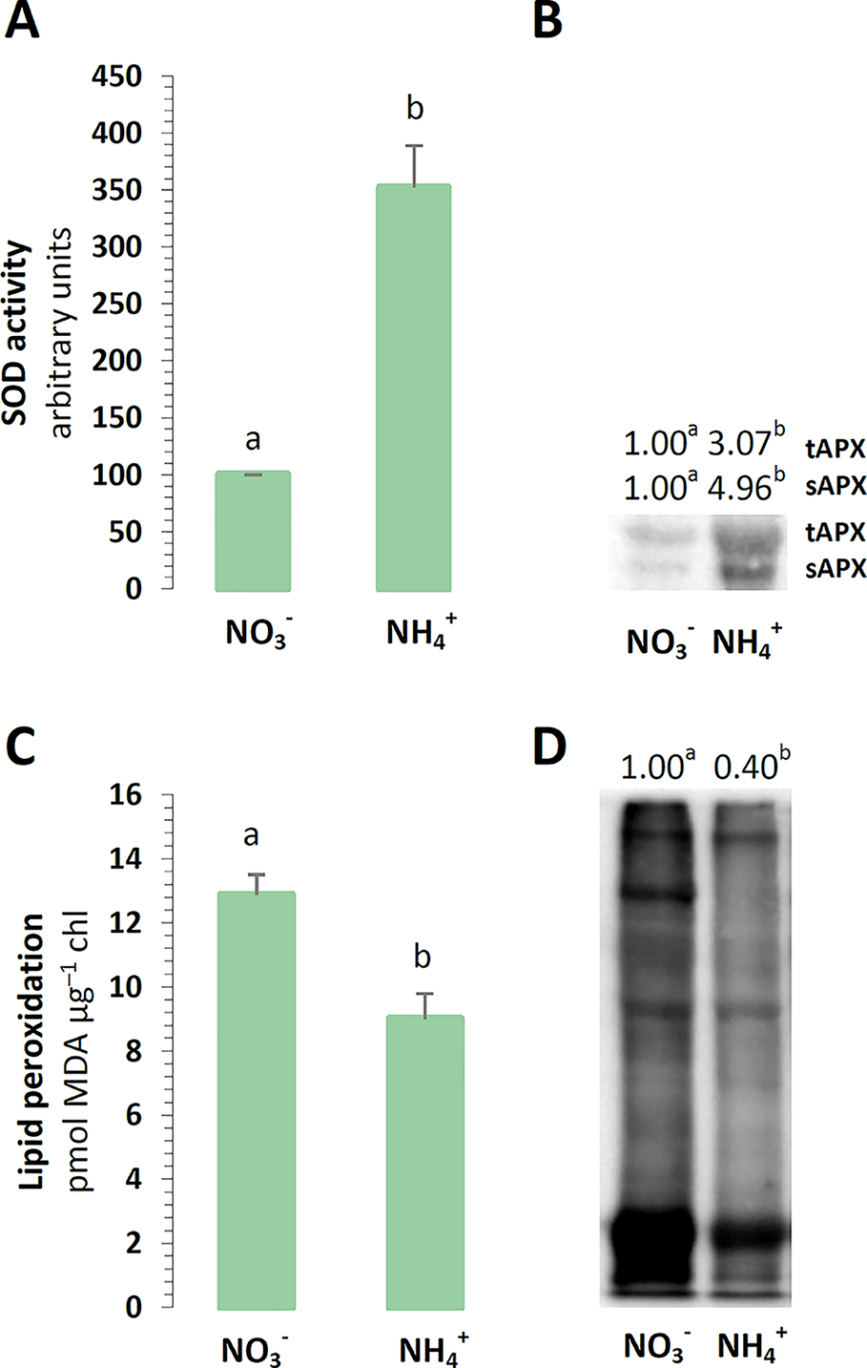
Defense towards reactive oxygen species (ROS) and oxidative stress markers in *Arabidopsis thaliana* nourished with 5 mM ${\mathrm{NH}}_{4}^{+}$ or 5 mM ${\mathrm{NO}}_{3}^{-}$ (control) as the only source of nitrogen. **(A)** Total activity of superoxide dismutase (SOD) in chloroplasts. **(B)** Protein abundance of thylakoid and stromal ascorbate peroxidase (tAPX, 38 kDa; sAPX, 33 kDa). **(C)** Lipid peroxidation in isolated chloroplasts. **(D)** Content of protein-bound carbonyls in chloroplasts. Representative immunoblots are shown out of n = 3−4 independent replicates. Bars or bands with different letters indicate significant difference (p ≤ 0.05).

We aimed to verify if ${\mathrm{NH}}_{4}^{+}$ nutrition is a stress factor to chloroplasts in *A. thaliana* grown with either ${\mathrm{NO}}_{3}^{-}$ or ${\mathrm{NH}}_{4}^{+}$ as the sole source of nitrogen. As the first step, the scavenging capacity of chloroplast antioxidants was determined. The major ROS produced in the chlETC might be O_2_˙^-^, and accordingly, the activity of SOD was higher in the plants nourished with ${\mathrm{NH}}_{4}^{+}$ (**Figure 1A**). The subsequent product of SOD activity should be H_2_O_2_, which would need to be further utilized. The protein levels of the thylakoid and stromal APX were higher in plants in the presence of ${\mathrm{NH}}_{4}^{+}$ (**Figure 1B**). If ROS production could not be balanced by the antioxidant systems, it could lead to tissue damage. The peroxidation levels of the membrane lipids were lower in the chloroplasts exposed to ${\mathrm{NH}}_{4}^{+}$ nutrition (**Figure 1C**). Similarly to the results of Podgórska et al. (2013), the carbonylation levels in the chloroplast proteins were generally lower upon ${\mathrm{NH}}_{4}^{+}$ nutrition (**Figure 1D**).

### Photosynthetic Pigment Levels

**Figure 2:**
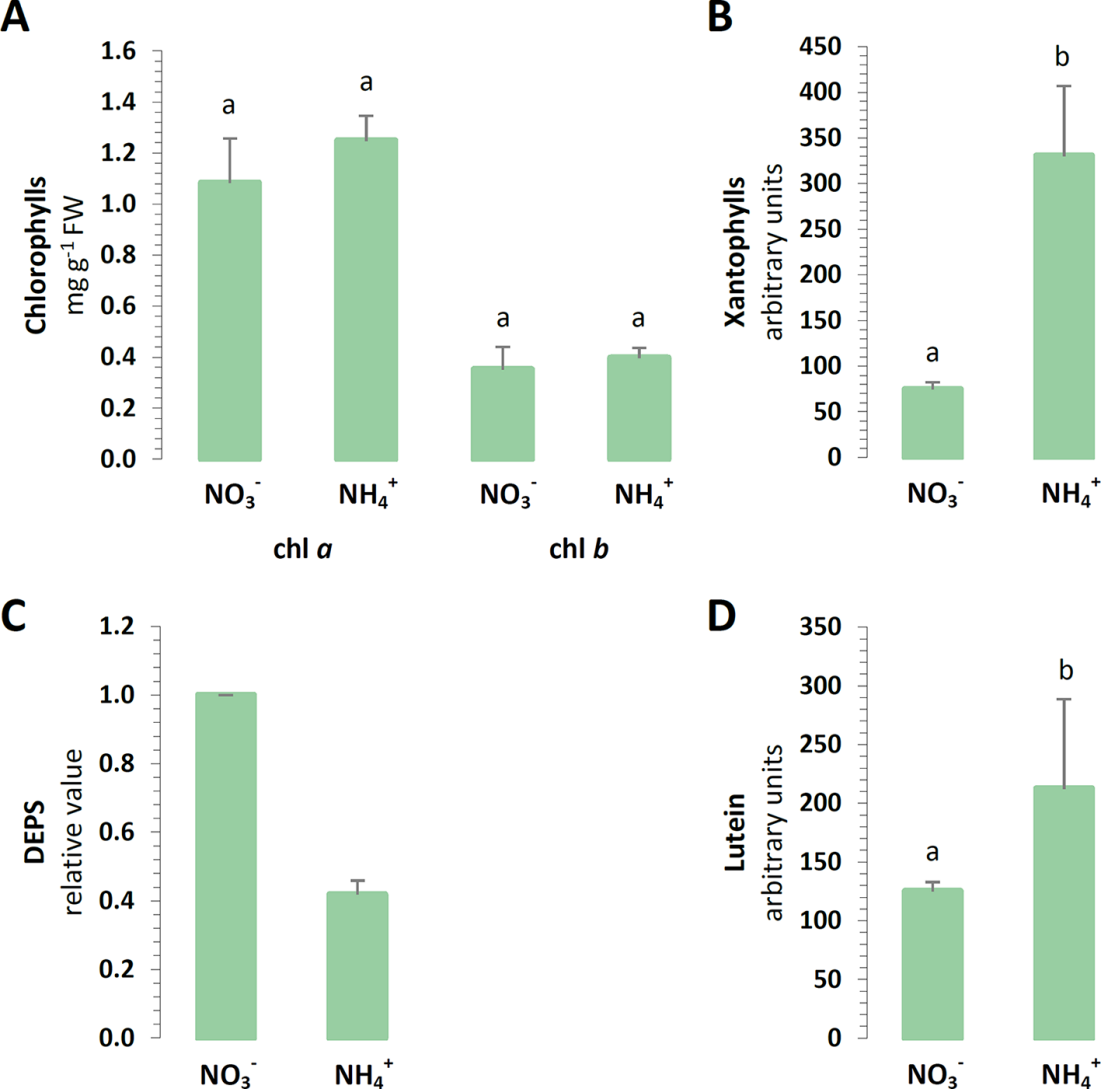
Foliar pigment levels in *A. thaliana* nourished with 5 mM ${\mathrm{NH}}_{4}^{+}$ or 5 mM ${\mathrm{NO}}_{3}^{-}$ (control) as the only source of nitrogen. **(A)** Levels of chlorophyll (chl) *a* and chl *b*. **(B)** Content of xanthophyll cycle pigments. **(C)** De-epoxidation state (DEPS) of xanthophylls. **(D)** Content of lutein. Bars with different letters indicate significant difference (p ≤ 0.05).

To carry on the identification of changes in chloroplast defense, the light absorbing antennae systems of the plants were analyzed. The chl *a* and chl *b* contents of the major pigments in the leaves were not affected by the nitrogen source (**Figure 2A**). Moreover, aside from absorbing and transferring light energy, carotenoids could also serve as photoprotective agents. A cycle of three carotenoids: violaxanthin, antheraxanthin, and zeaxanthin could additionally quench overexcitation energy in the chlETC. The content of all xanthophylls was induced in the plants nourished with ${\mathrm{NH}}_{4}^{+}$ (**Figure 2B**). Essentially, the de-epoxidation of violaxanthin to zeaxanthin could affect the efficiency of the xanthophyll cycle in photoprotection. Despite the higher total xanthophyll content, the de-epoxidation state (DEPS) was lower during ${\mathrm{NH}}_{4}^{+}$ nutrition (**Figure 2C**). Lutein, the major xanthophyll, which could act as a protective shield, was greater in plants nourished with ${\mathrm{NH}}_{4}^{+}$ (**Figure 2D**).

### Organization of the Photosynthetic Apparatus and Antenna Complexes

**Figure 3:**
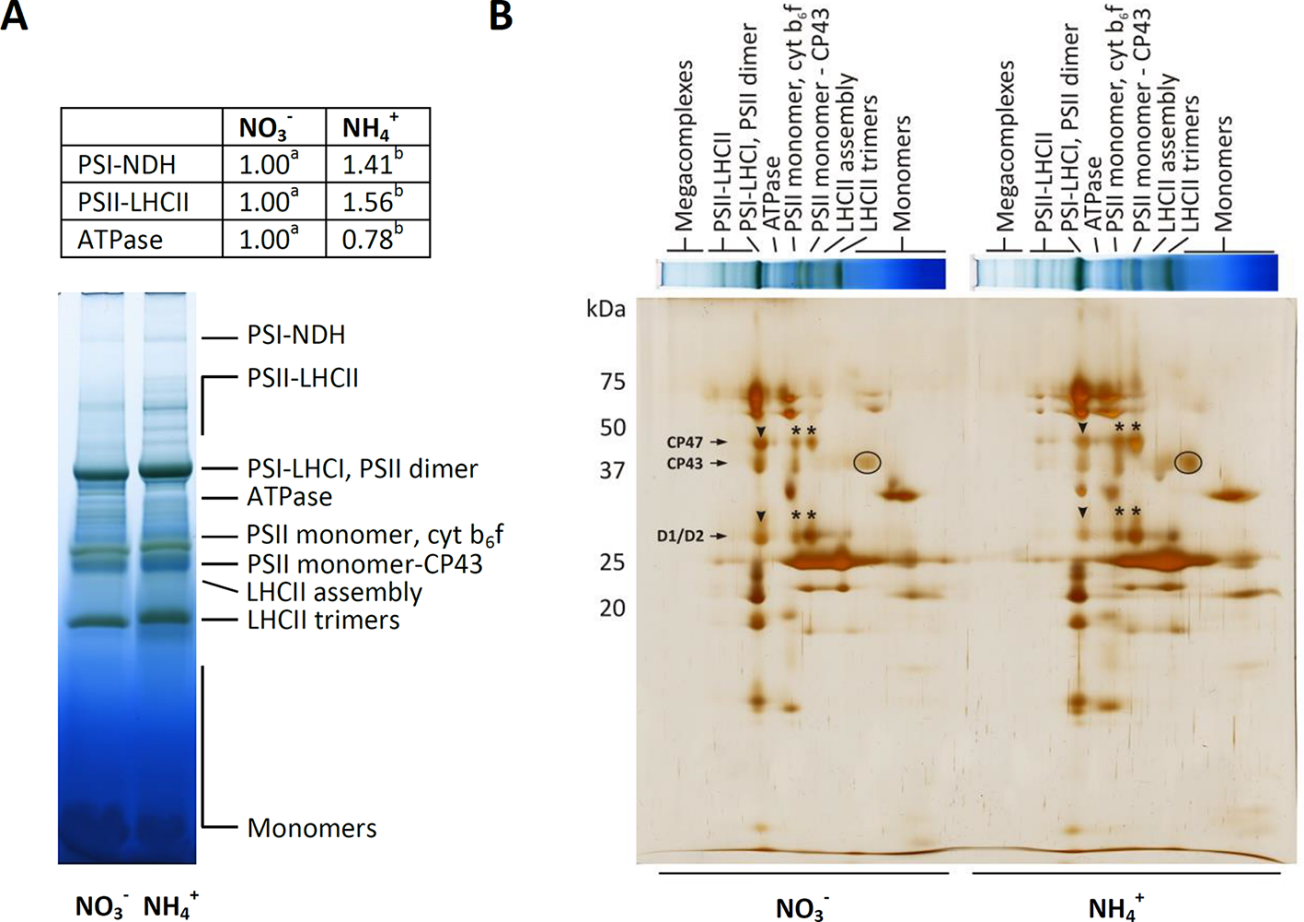
Composition of the photosynthetic protein complexes in *A. thaliana* nourished with 5 mM ${\mathrm{NH}}_{4}^{+}$ or 5 mM ${\mathrm{NO}}_{3}^{-}$ (control) as the only source of nitrogen. **(A)** Identification of thylakoid protein complexes in Blue Native (BN)-polyacrylamide gel electrophoresis (PAGE)-gel. Thylakoid membranes were solubilized in 1% dodecyl maltoside (DDM), and the protein extracts containing 8.3 µg of chlorophyll were separated by electrophoresis. Bands with different letters quantified by densitometry indicate significant difference (p ≤ 0.05). **(B)** Patterns of thylakoid protein complexes in second dimension by 2D-PAGE. BN-PAGE strips were denatured and separated on sodium dodecyl sulfate (SDS)-Urea-PAGE and were silver-stained. Representative gels are shown out of n = 3−6 independent replicates.

Photosynthetic light reactions take place in thylakoid membranes, and their efficiency could depend on the organization of multi-subunit protein complexes and light-harvesting complexes. The BN-PAGE gels containing equal amounts of chlorophyll per lane revealed that the distribution of the major bands was intensified during ${\mathrm{NH}}_{4}^{+}$ nutrition (**Figure 3A**). The band corresponding to megacomplexes showed significantly greater abundance of PSI-NDH in the thylakoids of the plants nourished with ${\mathrm{NH}}_{4}^{+}$ (**Figure 3A**). To a similar extent all bands that represent supercomplexes (PSII-LHCII) were of higher abundance in thylakoids of NH4+ grown plants (**Figure 3A**). The abundance of the megacomplexes and supercomplexes were very low in the control. The only band that was identified to be less pronounced in the thylakoids of the plants under ${\mathrm{NH}}_{4}^{+}$ nutrition was that of the ATPase complex (**Figure 3A**). To further characterize their abundances, the constituent subunit pattern of the thylakoid membrane protein complexes was analyzed after separation in the second dimension. The image of the 2D-SDS-PAGE proteins of the WT thylakoids showed a typical pattern observed in *A. thaliana* plants (**Figure 3B**). The thylakoid membranes were isolated after 3 h of light, so a PSII monomer without CP43 subunit was visible, indicating the activation of the PSII repair cycle. This band was more intensive in the thylakoids isolated from the plants under ${\mathrm{NH}}_{4}^{+}$ nutrition. Based on the 2D-PAGE, D1 and D2 proteins (around 30 kDa), and CP47 and CP43 proteins (around 47 and 43 kDa), were more abundant in PSII dimer fractions in the plants nourished with ${\mathrm{NO}}_{3}^{-}$ (**Figure 3B**, arrowheads), while in response to ${\mathrm{NH}}_{4}^{+}$ nutrition these proteins had higher abundance in PSII monomer and PSII monomer without CP43 fractions (**Figure 3B**, asterisks). Moreover, considerably higher amount of CP43 was also present in CP monomer fraction (**Figure 3B**, circle) in ${\mathrm{NH}}_{4}^{+}$ than in ${\mathrm{NO}}_{3}^{-}$ nourished plants. Taken together, these observations show that PSII repair cycle is more active under ${\mathrm{NH}}_{4}^{+}$ nutrition.

### Photosynthetic Capacity

**Figure 4:**
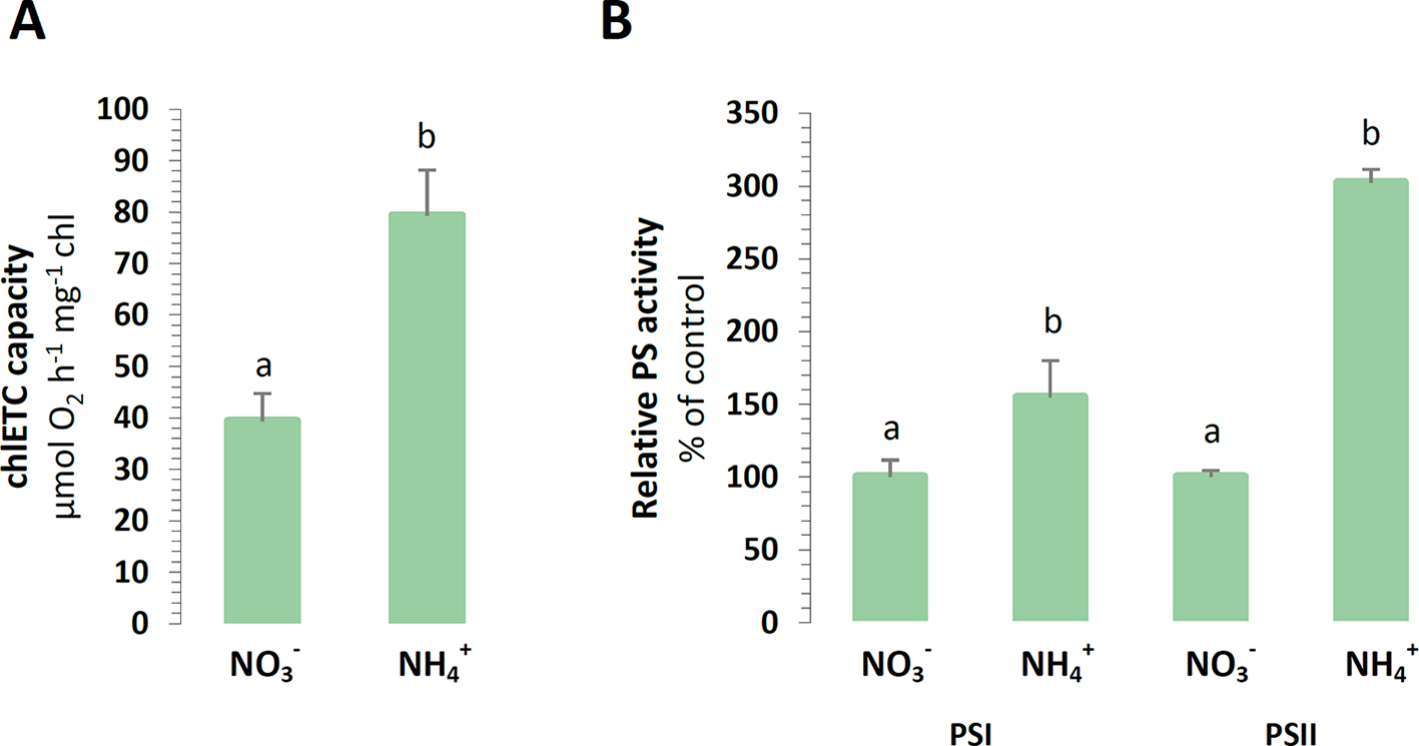
Capacity of chloroplast electron transport chain in *A. thaliana* nourished with 5 mM ${\mathrm{NH}}_{4}^{+}$ or 5 mM ${\mathrm{NO}}_{3}^{-}$ (control) as the only source of nitrogen. **(A)** Total electron transport chain capacity. **(B)** Capacity of Photosystem I (PSI) and Photosystem II (PSII). Bars with different letters indicate significant difference (p ≤ 0.05).

In order to estimate the functional state of the photosynthetic apparatus of the plants, the capacity of its components was measured *in vitro*. The efficiency of the chlETC was higher during ${\mathrm{NH}}_{4}^{+}$ nutrition (**Figure 4A**), and the activity of PSI and PSII in the presence of artificial electron acceptors was respectively induced under ${\mathrm{NH}}_{4}^{+}$ nutrition (**Figure 4B**).

**Figure 5:**
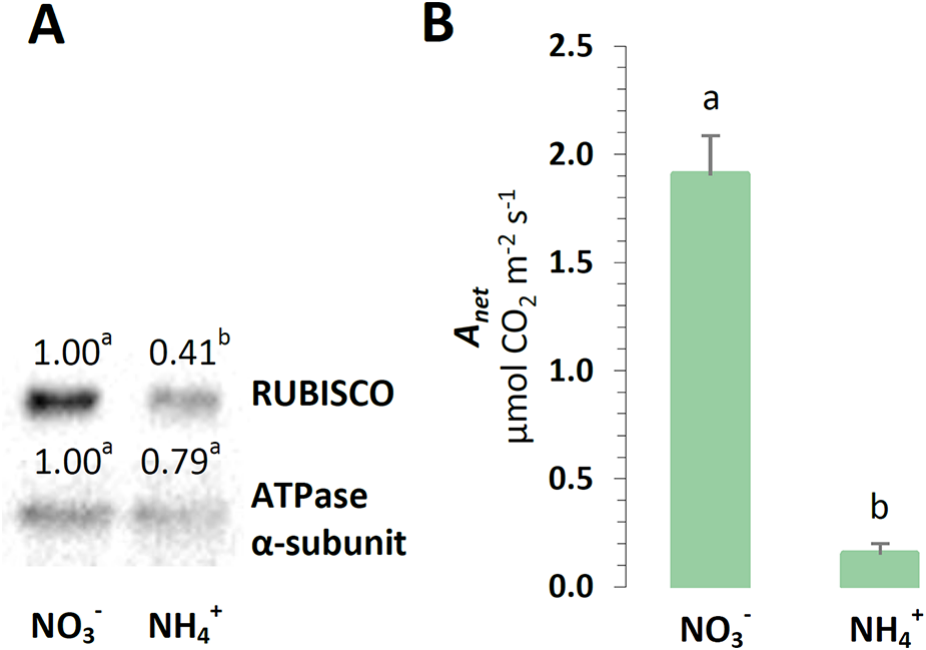
Photosynthetic performance in *A. thaliana* nourished with 5 mM ${\mathrm{NH}}_{4}^{+}$ or 5 mM ${\mathrm{NO}}_{3}^{-}$ (control) as the only source of nitrogen. **(A)** Protein abundance of Ribulose-1,5-bisphosphate-carboxylase/oxygenase (RUBISCO, 50 kDa) and ATPase α-subunit (α-ATPase, 55 kDa). Representative immunoblots are shown out of n = 3−4 independent replicates. **(B)** Photosynthesis net rate (A_net_). Bars or bands with different letters indicate significant difference (p ≤ 0.05).

To further examine the photosynthetic efficiency of the plants under ${\mathrm{NH}}_{4}^{+}$ nutrition, their photosynthetic yield was analyzed. During the final step of the photosynthetic light reactions, a chloroplast ATP-synthase could use the proton motive force generated by the chlETC to produce ATP. The protein level of the α-ATPase subunit was slightly lower in the plants treated with ${\mathrm{NH}}_{4}^{+}$, but this difference was not statistically significant (**Figure 5A**). The energy gained during photosynthetic light driven reactions could be used in the CBB cycle for carbon reactions. The photosynthetic CO_2_ assimilation rate was even 10 times lower in the plants nourished with ${\mathrm{NH}}_{4}^{+}$ (**Figure 5B**). The carboxylation reaction of photosynthesis could involve RUBISCO to facilitate CO_2_ fixation. A decrease in RUBISCO protein abundance was observed in the plants under ${\mathrm{NH}}_{4}^{+}$ nutrition (**Figure 5A**).

### Electron Routes in the chlETC

**Figure 6:**
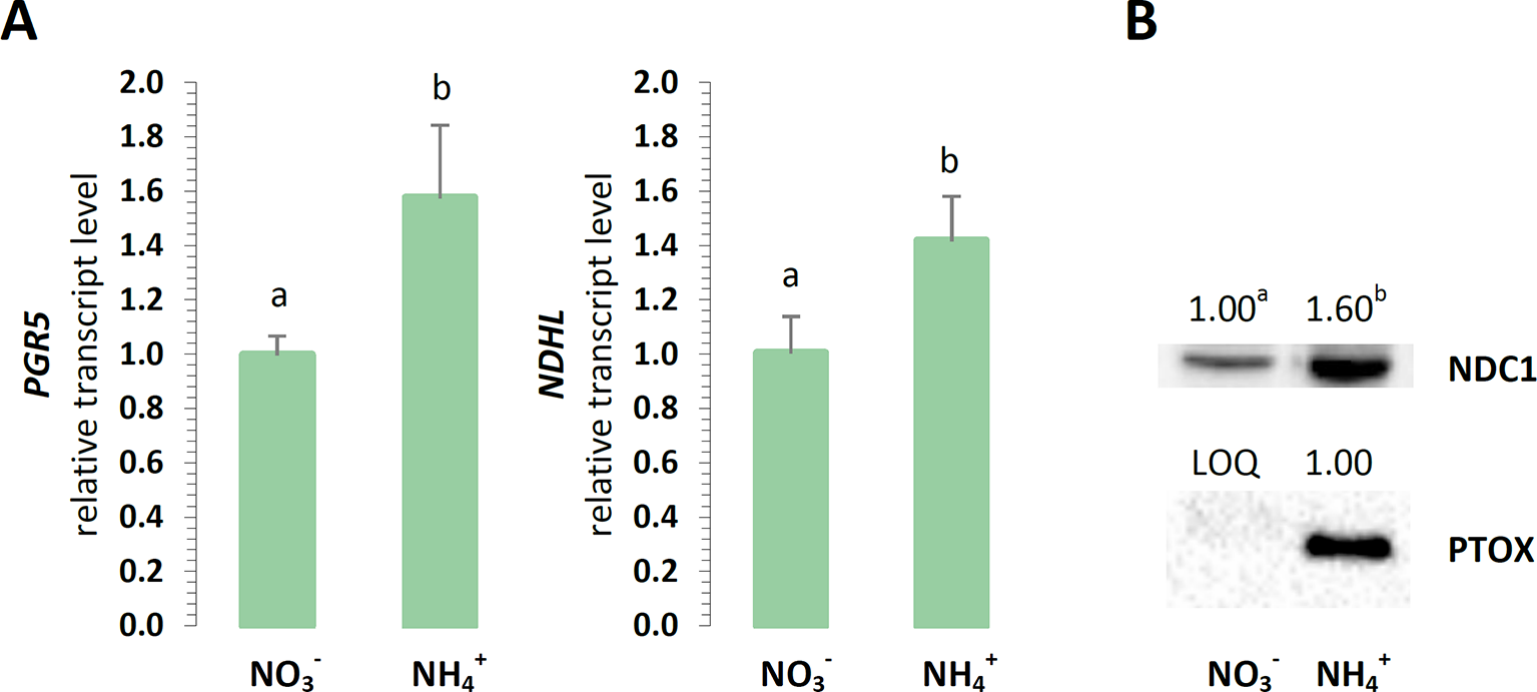
Electron dissipation from the chloroplast electron transport chain in *A. thaliana* nourished with 5 mM ${\mathrm{NH}}_{4}^{+}$ or 5 mM ${\mathrm{NO}}_{3}^{-}$ (control) as the only source of nitrogen. **(A)** Transcript levels of NAD(P)H:plastoquinone oxidoreductase subunit L (*NDHL*) and proton gradient regulation 5 (*PGR5*). The relative expression was set to 1 in WT plants grown with ${\mathrm{NO}}_{3}^{-}$ (control) for reference. **(B)** Protein abundance of NAD(P)H dehydrogenase C1 (NDC1, 51 kDa) and plastid terminal oxidase (PTOX, 37 kDa; LOQ, under limit of quantification). Representative immunoblots are shown out of n = 3−4 independent replicates. Bars or bands with different letters indicate significant difference (p ≤ 0.05).

For their optimal energy production, the chloroplasts could branch their electron routes to recycle electrons without the accumulation of reductive power. The engagement of cyclic electron transport around PSI was initially focused on, considering that energy is produced in these reactions without NADPH accumulation. The expression of *PGR5* was induced in relation to ${\mathrm{NH}}_{4}^{+}$ nutrition (**Figure 6A**), and also the expression of *NDHL* was upregulated in the plants treated with ${\mathrm{NH}}_{4}^{+}$ (**Figure 6A**). Furthermore, a type II NAD(P)H-dependent quinone oxidoreductase that is associate with plastoglobules could reduce the PQ pool in chloroplasts. The protein abundance of the chloroplast NDC1 was higher in the plants under ${\mathrm{NH}}_{4}^{+}$ nutrition (**Figure 6B**). Another protective function associated with PTOX could be accepting electrons from PQ, thereby competing with the chlETC. PTOX could reduce the number of electrons available for the photosynthetic electron flow, and therefore, this chlororespiratory activity would be required upon the fluctuation of the reduction status of the chloroplast. The protein levels of PTOX incrementally increased in the plants nourished with ${\mathrm{NH}}_{4}^{+}$ (**Figure 6B**).

### Pathways Engaged in the Export of Reductants Out of Chloroplasts

**Figure 7:**
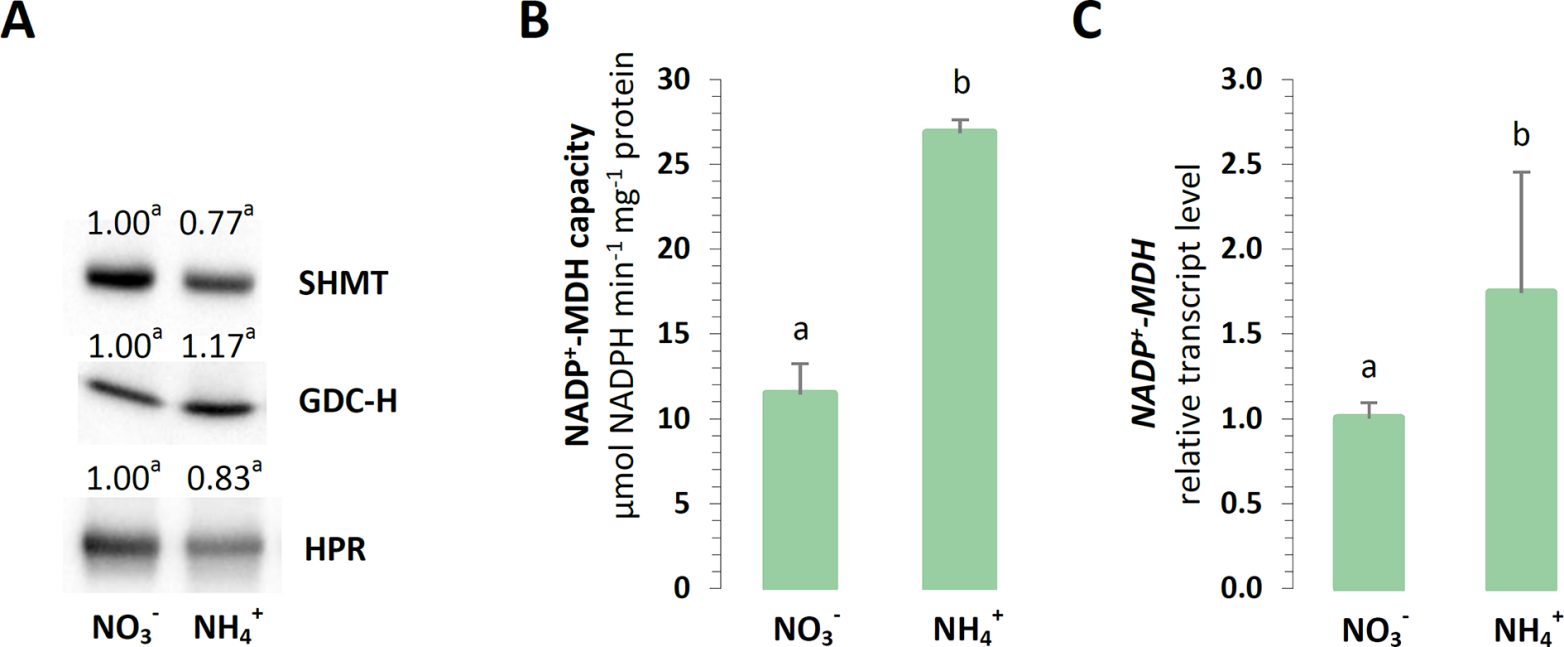
Export of reductants out of chloroplasts in *A. thaliana* nourished with 5 mM ${\mathrm{NH}}_{4}^{+}$ or 5 mM ${\mathrm{NO}}_{3}^{-}$ (control) as the only source of nitrogen. **(A)** Protein abundance of mitochondrial serine hydroxymethyltransferase (SHMT, 53 kDa); mitochondrial glycine decarboxylase complex H subunit (GDC-H, 16 kDa); and peroxisome hydroxypyruvate reductase (HPR, 43 kDa). Representative immunoblots are shown out of n = 3−4 independent replicates. **(B)** Capacity of NADP-malate dehydrogenase (NADP-MDH). **(C)** Transcript level of chloroplast *NADP-MDH*. The relative expression is set to 1 in WT plants grown with ${\mathrm{NO}}_{3}^{-}$ (control) for reference. Bars or bands with different letters indicate significant difference (p ≤ 0.05).

It has been postulated that the photorespiratory cycle not only recovers carbon, but it also mediates the partitioning of metabolites between chloroplast peroxisomes and mitochondria and facilitates the net oxidation of NADPH in the process. Nevertheless, the analyzed enzymes related to photorespiration were unaffected in the plants grown on different nitrogen sources. No changes in the protein levels of mitochondrial GDC-H, SHMT, or peroxisomal HPR were detected during ${\mathrm{NH}}_{4}^{+}$ nutrition (**Figure 7A**).

Another enzyme responsible for the direct export of reductive power out of chloroplasts could be NADP-MDH, so it was investigated whether the electron flow through the OAA/Mal valve would lead to reductant dissipation in the mitochondria. The maximal enzyme activity and expression of chloroplast *NADP-MDH* was strongly upregulated in all the plants nourished with ${\mathrm{NH}}_{4}^{+}$ (**Figures 7B, C**). Hence, the activity of the other forms of NAD-MDH was vital to the drainage of electrons into the mitochondria, as it was induced in the plants during ${\mathrm{NH}}_{4}^{+}$ nutrition (**Supplementary Figure 8B**).

### Efficiency of Photosynthesis and NPQ

**Figure 8:**
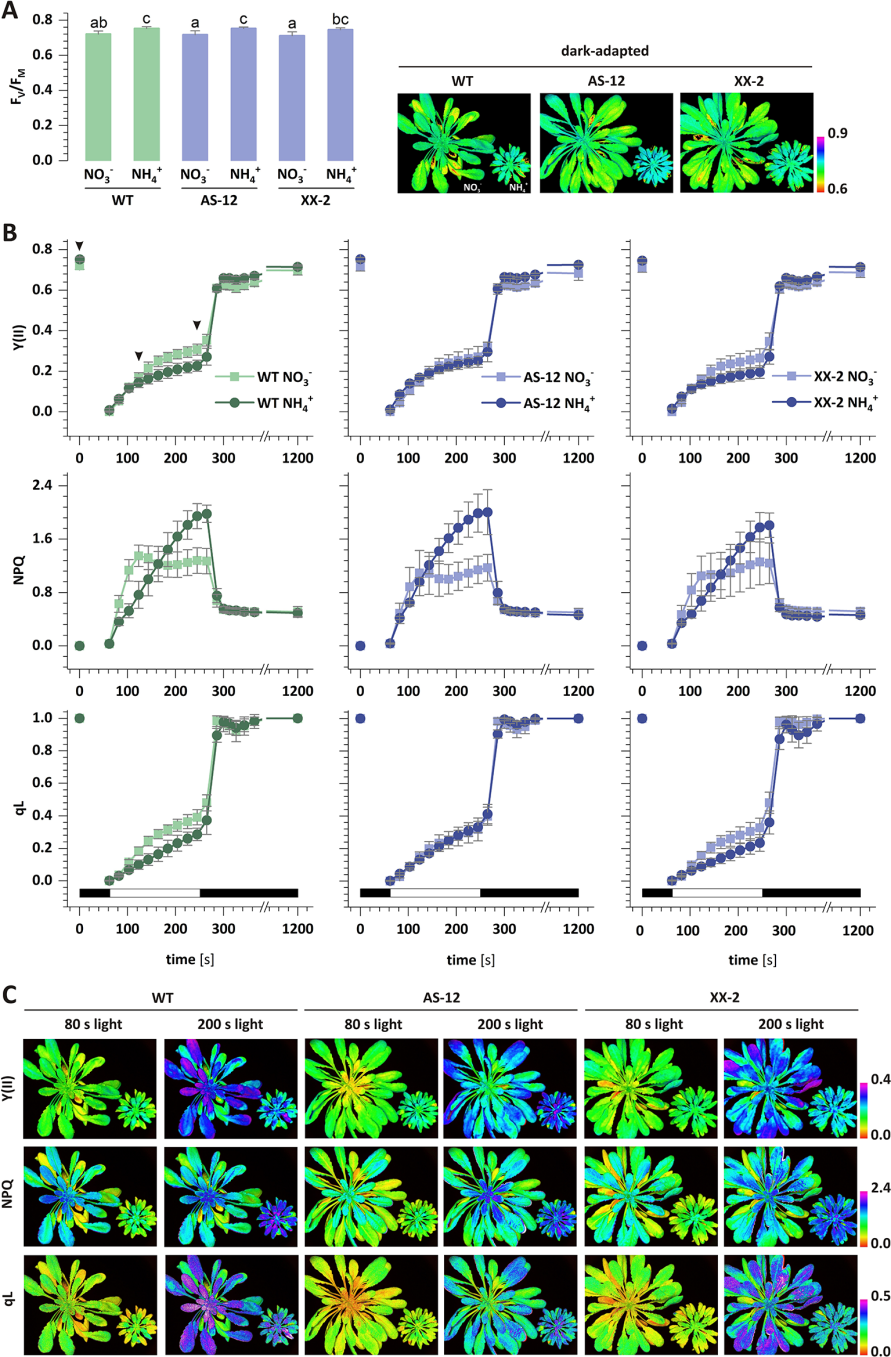
The efficiency of photosynthetic light reactions in transgenic *AOX1a* (antisense, AS-12 or overexpression, XX-2) or WT plants nourished with 5 mM ${\mathrm{NH}}_{4}^{+}$ or 5 mM ${\mathrm{NO}}_{3}^{-}$ (control) as the only source of nitrogen. Photosynthetic parameters: **(A)** F_V_/F_M_; and **(B)** Y(II), non-photochemical quenching (NPQ) and qL were measured during actinic light illumination of dark-adapted plants, followed by a dark recovery phase. **(C)** Images represent the distribution of the respective parameter values in *A. thaliana* rosettes in two time points: 80 s and 200 s of actinic illumination and in dark-adapted state (only for F_V_/F_M_). Selected time points are indicated by arrowhead in panel B plot. Black and white bars on plots represent dark and illumination phases of the measurement, respectively. Data are mean values ± SD from at least eight leaves. Results with different letters indicate significant difference (p ≤ 0.05).

The photochemistry of PSII was measured *in vivo* in the dark-adapted leaves of all the plants. The maximal quantum yield of PSII (F_V_/F_M_) was marginally increased in the WT plants nourished with ${\mathrm{NH}}_{4}^{+}$, compared to those under ${\mathrm{NO}}_{3}^{-}$ nutrition (**Figure 8A**). To further explore the role of AOX1a in chloroplast functioning under ${\mathrm{NH}}_{4}^{+}$ nutrition, the photosynthetic performance of the transgenic plants was analyzed. In the AS-12 and XX-2 mutants, the F_V_/F_M_ values were minimally increased in the plants nourished with ${\mathrm{NH}}_{4}^{+}$, compared to those under ${\mathrm{NO}}_{3}^{-}$ nutrition (**Figure 8A**). In the dark-adapted WT plants nourished with ${\mathrm{NO}}_{3}^{-}$ and illuminated with actinic light of high intensity, the effective quantum yield of PSII-Y(II) gradually increased, whereas in those under steady-state light conditions at the end of illumination, the quantum yield reached approximately 0.3, which was higher than 0.23 in the plants nourished with ${\mathrm{NH}}_{4}^{+}$ (**Figure 8B**). After the dark recovery phase of WT plants, the Y(II) parameter reaches a higher value under ${\mathrm{NH}}_{4}^{+}$ nutrition (**Figures 8B, C**), which corresponds to higher F_V_/F_M_ values, than in the control (**Figure 8A**). In the AS-12 mutant, there was no significant difference in the time course of Y(II) during illumination in both treatments, but the values of the steady-state Y(II) reached around 0.2 (**Figure 8B**). The response of the XX-2 mutant in both the ${\mathrm{NO}}_{3}^{-}$ or ${\mathrm{NH}}_{4}^{+}$ conditions was similar to those of the WT plants, but the Y(II) values in the XX-2 mutant were lower than those in the WT plants (**Figure 8B**).

Given that chlorophyll fluorescence could reflect the photoprotection-related effects in chloroplasts, the NPQ of the excess light energy that was absorbed could discriminate between AOX1a antisense and an overexpressor mutants grown on different nitrogen sources. The NPQ value in the WT plants nourished with ${\mathrm{NO}}_{3}^{-}$ rapidly increased to its maximum and remained stable towards the end of illumination (**Figure 8B**). In contrast, in the plants nourished with ${\mathrm{NH}}_{4}^{+}$, the rise in NPQ was more gradual, compared to those under ${\mathrm{NO}}_{3}^{-}$ nutrition, but the increase lasted throughout the illumination. Moreover, in the steady-state conditions, the NPQ values of the plants under ${\mathrm{NH}}_{4}^{+}$ nutrition were 50% higher than those of the plants during ${\mathrm{NO}}_{3}^{-}$ nutrition (**Figure 8B**). After the dark recovery phase, the NPQ values were still the same, indicating that the photoinhibition of PSII was similar in both treatments. In contrast to the corresponding results in the WT plants, there was no difference in the initial phase of the NPQ increase in the AS-12 mutant in both treatments. Furthermore, the NPQ values under steady-state conditions were similar to those observed in the WT plants (**Figure 8B**). The NPQ changes in the XX-2 mutant were similar to those in the WT plants in both treatments (**Figure 8B**). The photochemical quenching parameter (qL) in the WT plants nourished with ${\mathrm{NO}}_{3}^{-}$ was higher than in those with ${\mathrm{NH}}_{4}^{+}$ nutrition, and after the dark recovery phase, the qL value was fully restored (**Figure 8B**). There was no difference in the qL of the AS-12 mutant in both treatments, whereas the qL values of the XX-2 mutant were similar to those of the WT plants (**Figure 8B**).

### Phenotype and Oxidative Stress Markers During the Overexpression or Suppression of AOX1a

**Figure 9:**
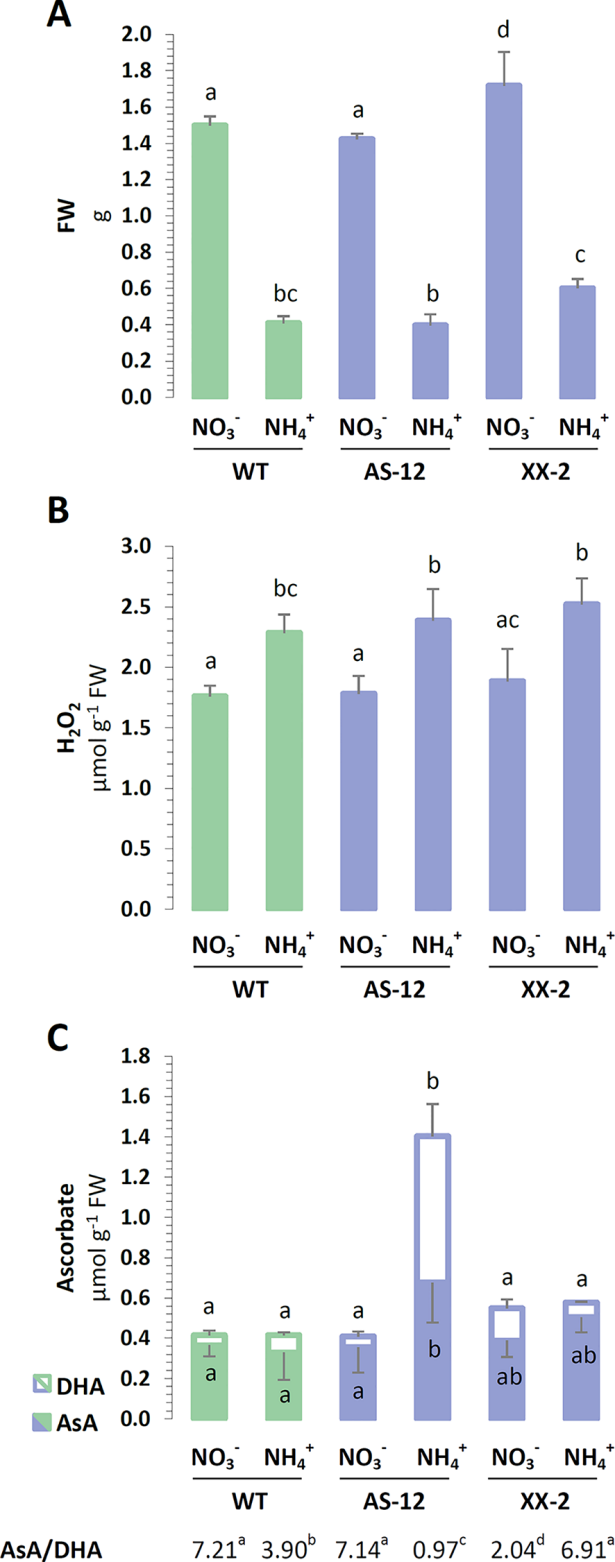
Characterization of *A. thaliana* after 8 weeks of hydroponics culture with 5 mM ${\mathrm{NO}}_{3}^{-}$ or 5 mM ${\mathrm{NH}}_{4}^{+}$ as the sole nitrogen source. AOX1a suppressing line (AS-12) and overexpressing line (XX-2) were derived from the presented wild type (WT, Col-0) plants. **(A)** Fresh weight (FW) of rosettes on respective nitrogen sources. **(B)** Hydrogen peroxide (H_2_O_2_) content of leaves. **(C)** Concentration of reduced ascorbate (AsA) and dehydroascorbate (DHA) as well as the derived AsA/DHA ratio. Bars with different letters indicate significant difference (p ≤ 0.05).

Considering that AOX is crucial to the redox regulation of photosynthetic organisms (Noguchi and Yoshida, 2007; Rasmusson et al., 2008), the occurrence of ${\mathrm{NH}}_{4}^{+}$ nutrition-related oxidative stress was analyzed in the plant tissues modified for the protein abundance of AOX1a (**Supplementary Figure 1**). The growth of WT plants was generally reduced when ${\mathrm{NH}}_{4}^{+}$ was used as the sole nitrogen source, compared to when ${\mathrm{NO}}_{3}^{-}$ was utilized. The rosette size and FW of the antisense AOX1a mutants were unchanged in the WT plants in both conditions, while the overexpression of AOX1a resulted in improved growth parameters (**Figure 9A**). The FW of XX-2 mutants was approximately 15% higher when they were nourished with ${\mathrm{NO}}_{3}^{-}$ and even 50% higher under ${\mathrm{NH}}_{4}^{+}$ nutrition, compared to the FW of the WT plants.

To assess the impact of AOX1a on the occurrence of stress in the plants in response to ${\mathrm{NH}}_{4}^{+}$ nutrition, their ROS and antioxidant levels were determined. ${\mathrm{NH}}_{4}^{+}$ nutrition augmented the H_2_O_2_ content of the leaf tissues in all genotypes (**Figure 9B**). The major low-mass antioxidant is ascorbate, and the total ascorbate pool was substantially enlarged in the AS-12 mutant plants nourished with ${\mathrm{NH}}_{4}^{+}$ (**Figure 9C**). The increase in ascorbate was mainly due to higher DHA content, consequently the ratio of AsA to DHA was lower in the WT plants and was even more reduced in the AS-12 mutant plants when they were nourished with ${\mathrm{NH}}_{4}^{+}$ (**Figure 9C**). Compared to the WT plants, the XX-2 mutant had an oxidized ascorbate redox state when growing on NO_3-_. These findings suggest that the ascorbate pool is oxidized when plants are challenged with the dysfunction of AOX1a and under ${\mathrm{NH}}_{4}^{+}$ nutrition, whereas the overexpression of AOX1a improves the resistance of the plants to ${\mathrm{NH}}_{4}^{+}$ nutrition. The metabolic differences between AOX1a mutants and WT plants were not pronounced; the full results of the photosynthetic dependent parameters are shown in the **Supplementary Materials**.

## Discussion

### Ammonium Nutrition Leads to Perturbation of the Oxidation-Reduction Status of Chloroplasts But Does Not Result in the Oxidative Damage of the Biomolecules in These Organelles

Considering that ${\mathrm{NH}}_{4}^{+}$ as the nitrogen source was expected to cause the overreduction of the chlETC. In our previous study, prolonged ${\mathrm{NH}}_{4}^{+}$ nutrition increased the redox state of *Arabidopsis* leaf tissue (Podgórska et al., 2013) and consequently increased the ROS level in leaves (Podgórska et al., 2013; Podgórska et al., 2015). Nevertheless, the oxidative damage of biomolecules occurred in the mitochondria (Podgórska et al., 2013) but not in the chloroplasts under long-term ${\mathrm{NH}}_{4}^{+}$ nutrition. In the present study the chloroplasts were characterized by lower abundance of oxidized proteins or by lesser lipid peroxidation (**Figures 1C, D**), hence, oxidative stress did not occur in the chloroplasts in response to ${\mathrm{NH}}_{4}^{+}$ nutrition. It can be achieved by limiting the chloroplast ROS production or by enhancing the antioxidant system capacity or by both. Moreover, the O_2_˙^-^ derived from the Mehler reaction is eventually converted into water due to the activity of the chloroplast-associated SOD and APX, closing the water-to-water cycle, which is also named as pseudo-cyclic electron transport chain (Shikanai and Yamamoto, 2017). There is no clear data concerning chloroplastic ROS production under ${\mathrm{NH}}_{4}^{+}$ nutrition, but Zhu et al. (2000) and Gerendás et al. (1997) substantiated the stimulation of the Mehler reaction in plants nourished with ${\mathrm{NH}}_{4}^{+}$. In our current study, the activity of SOD was stimulated in the chloroplasts of the plants under ${\mathrm{NH}}_{4}^{+}$ nutrition (**Figures 1A, B**). SOD has to cooperate with other enzymatic H_2_O_2_ scavengers to totally reduce ROS in Mehler-peroxidase reactions (Foyer and Harbinson, 1994), the protein levels of thylakoid and stromal APX were also augmented in the chloroplasts of the plants nourished with ${\mathrm{NH}}_{4}^{+}$ (**Figure 1B**). Nonetheless, we previously demonstrated that long-term growth of *A. thaliana* did not affect the activity of the chloroplast-localized ascorbate recycling enzymes dehydroascorbate reductase (DHAR) and monodehydroascorbate reductase (MDHAR) (Podgórska et al., 2013). Another H_2_O_2_-responsive antioxidant in chloroplasts could be peroxiredoxin Q (PRXQ) whose protein level was found to be higher in *A. thaliana* under ${\mathrm{NH}}_{4}^{+}$ nutrition (Podgórska et al., 2018). Unfortunately, we were not able to measure ascorbate directly in isolated chloroplasts, which is one of the most important antioxidants. Ascorbate is not only needed in the Mehler-peroxidase reactions, but it is also a substrate for violaxanthin de-epoxidase (VDE) in xanthophyll cycle. The concentration of other water-soluble antioxidants such as glutathione was increased in the chloroplasts under ${\mathrm{NH}}_{4}^{+}$ nutrition (Podgórska et al., 2013). Considering that plants need to immediately detect the changes in their redox status to prevent further damage in their chloroplasts, thioredoxins had been proposed as redox sensors and transmitters. The up-regulation of chloroplast NADPH-dependent thioredoxin reductase C (NTRC), and the Fd-dependent thioredoxins TRXx or TRXy2 in the ${\mathrm{NH}}_{4}^{+}$-nourished *A. thaliana* (Podgórska et al., 2018) could suggest them to be important mediators in redox signaling.

### An Increase in Energy-Dependent NPQ and the Upregulation of the Cyclic chlETC Ensure the chlETC Functioning Under ${\mathrm{NH}}_{4}^{+}$ Nutrition

Plants have developed a network of adaptive and protective mechanisms through the regulation of electron transport in the chlETC and dissipation of energy absorbed by photosynthetic pigments. Higher chlETC capacity (**Figure 4A**) and enhanced activity of PSI and PSII *in vitro* (**Figure 4B**) were observed during the exclusive nutrition of the plants with ${\mathrm{NH}}_{4}^{+}$, suggesting that no damage of the photosystems was induced by ${\mathrm{NH}}_{4}^{+}$ stress. However, this observation did not necessarily reflect the functioning of the photosystems *in vivo* when all the protective and adaptive mechanisms were activated. The BN-PAGE analysis of the thylakoid-localized complexes revealed that the abundance of the PSII-LHCII complexes was greater in the plants nourished with ${\mathrm{NH}}_{4}^{+}$ (**Figure 3A**). Moreover, the subtle differences in the migration of bands corresponding to PSI, PSII dimer (**Figure 3A**) and the variations in the abundance of spots corresponding to specific proteins (**Figure 3B**) signify that ${\mathrm{NH}}_{4}^{+}$ nutrition induces structural modification in photosynthetic apparatus. Nevertheless, further analysis on this is needed. PSII is considered as the photosystem primarily exposed to photodamage (Baker, 2008), so an analysis of chl *a* fluorescence kinetics can display the functional state of PSII *in vivo*. Our results showed that the marginally higher value of the maximal quantum yield of PSII in the ${\mathrm{NH}}_{4}^{+}$-nourished plants (**Figure 8B**) corresponded to the upregulated activity of PSII *in vitro*, but the curves illustrating the parameters related to the usage of absorbed energy for photochemical reactions indicated that the processes were significantly downregulated in the ${\mathrm{NH}}_{4}^{+}$-nourished plants during light period (**Figures 8B, C**). The NPQ values were initially high in the plants nourished with ${\mathrm{NO}}_{3}^{-}$, but they became eventually higher in the plants responding to ${\mathrm{NH}}_{4}^{+}$ nutrition (**Figure 8B**). Whereas the value for qI is unchanged during ${\mathrm{NH}}_{4}^{+}$ nutrition (**Supplementary Figure 9A**), suggesting that PSII photoinhibitory quenching is not induced. Findings on *Phaseolus vulgaris* grown with ${\mathrm{NH}}_{4}^{+}$ refute the occurrence of photoinhibition (Zhu et al., 2000). The photoinhibition of PSII only occurs if the rate of damage exceeds the rate of repair. Alencar et al. (2019) demonstrated that in rice supplied with ${\mathrm{NH}}_{4}^{+}$, the abundance of protein D1 was unchanged. Based on BN-PAGE profiles (**Figure 3B**), the activity of D1 repair cycle may be increased in ${\mathrm{NH}}_{4}^{+}$-nourished plants. Moreover, the PSII monomer without CP43 subunit in the plants under ${\mathrm{NH}}_{4}^{+}$ nutrition (**Figure 3B**) was more abundant, validating the possibly higher activity of PSII repair cycle.

**Figure 10:**
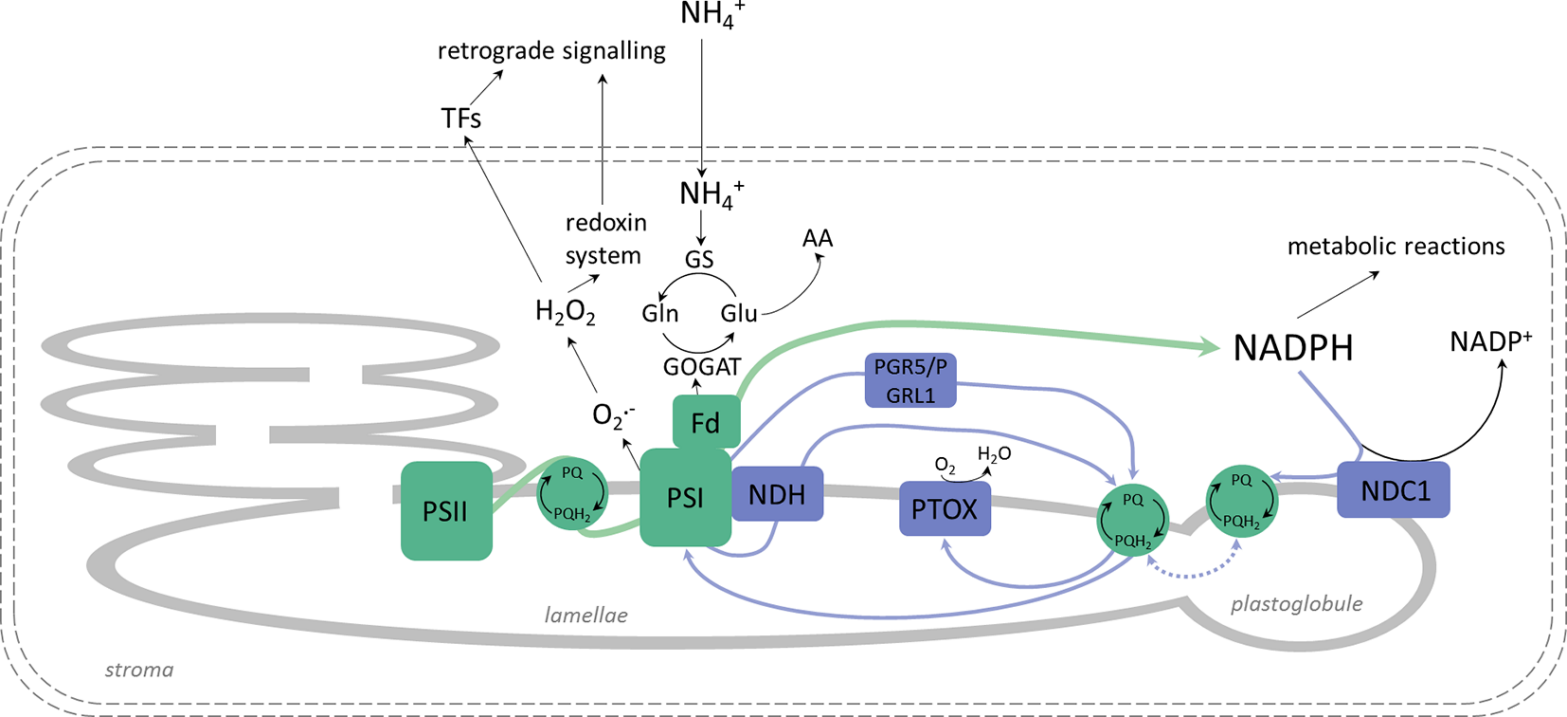
Changes in chlETC functioning, linear electron transport (LET; green arrows) route and alternative electron pathways (blue arrows) under ${\mathrm{NH}}_{4}^{+}$ nutrition. The inevitable activity of the glutamine synthetase - glutamine:2-oxoglutarate aminotransferase (GS-GOGAT) cycle consumes electrons from ferredoxin (Fd) for ${\mathrm{NH}}_{4}^{+}$ assimilation into amino acids (AA). Higher NAD(P)H dehydrogenase C1 (NDC1) protein abundance in plastoglobules transfers electrons from NADPH to plastoquinone (PQ), balancing the stromal redox pool. Consequently, less free PQ are available for cyclic electron transport (CET), and the PQ pool is more reduced in grana lamellae. Electrons from photosystem I (PSI) enter pseudocyclic electron transport (water-to-water cycle), which may lead to reactive oxygen species (ROS) production in chloroplasts. H_2_O_2_ may be engaged in signaling, and plastid terminal oxidase (PTOX) may act as the positive regulator of chloroplast to nucleolus retrograde signaling or communication with the alternative oxidase (AOX).

${\mathrm{NH}}_{4}^{+}$ nutrition extensively increased the qE quenching parameter in *A. thaliana*, indicating that excitation energy was lost as heat (**Supplementary Figure 9B**). Apart from LET, the specific role of CET and pseudocyclic electron transport in the generation of ΔpH needed for the induction of qE has been strongly postulated. In our current study, ${\mathrm{NH}}_{4}^{+}$ nutrition induced both CET branches as it was affirmed by the increased expression of *PGR5* (**Figure 6A**), by the significantly greater abundance of NDH-PSI supercomplex (**Figure 3A**), and by *NDHL* expression (**Figure 6A**), all together creating an safety valve for PSI (**Figure 10**). Besides being induced by the rise of ΔpH across the thylakoid membrane *via* the protonation of the PSII subunit S protein (PsbS), qE is also dependent on the metabolism of xanthophyll pigments (Ware et al., 2014 and references therein). The formation of photoprotective states in higher plants possibly requires a structural reorganization of the photosynthetic membranes involving the dissociation of LHCII from PSII promoted by de-epoxidation of violaxanthin to zeaxanthin (Johnson et al., 2011). In addition, the formation of qZ is strictly dependent on zeaxanthin but independent of PsbS (Liu et al., 2019). The relaxation of qZ depends on zeaxanthin epoxidation, and it is linked to the kinetics of the zeaxanthin pool. Pools of carotenoids involved in the xanthophyll cycle were extensively augmented under ${\mathrm{NH}}_{4}^{+}$ nutrition (**Figure 2B**), so the changes in qZ were also possibly induced. In contrast to this, zeaxanthin production was reduced in *P. vulgaris* grown in the presence of ${\mathrm{NH}}_{4}^{+}$ (Bendixen et al., 2001). The role and mechanism of the carotenoid-dependent component of NPQ have to be further studied, considering that ${\mathrm{NH}}_{4}^{+}$ nutrition resulted in higher zeaxanthin level but lower de-epoxidation states (DEPS) of the xanthophyll cycle pigments (**Figure 2C**). Furthermore, the high lutein levels during ${\mathrm{NH}}_{4}^{+}$ nutrition substantiated the importance of carotenoids in *Arabidopsis* under ${\mathrm{NH}}_{4}^{+}$ nutrition (**Figure 2D**).

### The Upregulated CET/NDC1—PTOX Pathways Under ${\mathrm{NH}}_{4}^{+}$ Nutrition May Function as an Efficient Membrane-Localized Reductant Sink in Chloroplasts

In higher plants, PTOX is responsible for the carotenoid biosynthesis during the developmental processes in chloroplasts (Krieger-Liszkay and Feilke, 2016). Nevertheless, its presence in mature tissues suggests that PTOX may also be involved in other processes: chlororespiration and light-independent oxygen consumption in the chloroplasts, permitting plastoquinol oxidation during the dark period (Nawrocki et al., 2019); regulation of the CET pathway (Joët et al., 2002); and/or protection from oxidative stress (Stepien and Johnson, 2009). During ${\mathrm{NH}}_{4}^{+}$ nutrition PQH_2_-associated with CET activity may be a natural substrate for PTOX activity. Such hypothesis was confirmed by the enormous increase in the PTOX protein levels (**Figure 6B**) and CET elements (**Figure 6A**) under ${\mathrm{NH}}_{4}^{+}$ nutrition in our current study. Moreover, NDC1 activity also influences the redox state of the plastoglobule-localized PQ pool (van Wijk and Kessler, 2017), and considering that plastoglobules are integral with the edges of the stroma lamellae (Fatihi et al., 2015), it can be assumed that both the plastoglobule and stroma lamellae PQ subpools mix together. Therefore, we suggest that non-photochemical PQ reduction due to NDC1 activity is a pathway that is naturally parallel to other light-dependent PQ-reducing processes in thylakoids and that NDC1 activity can be recognized as an alternative sink pathway that balances the excess chloroplastic NADPH under ${\mathrm{NH}}_{4}^{+}$ nutrition. The possible alternative electron route consistent of NDC1 together with PTOX can function as electron dissipation when plants are nourished with ${\mathrm{NH}}_{4}^{+}$ (**Figure 10**). Such hypothesis is corroborated by the markedly upregulated protein levels of NDC1 in the plants under ${\mathrm{NH}}_{4}^{+}$ nutrition (**Figure 6B**). Remarkably, PTOX is considered an important component of redox sensing in higher plants in relation to the redoxin system (Kambakam et al., 2016; Feilke et al., 2017; and references therein), which regulates several chloroplast-localized processes including PSI assembly (Zhu et al., 2016) and improves the tolerance of plants to stress (Johnson and Stepien, 2016).

### Redox Equivalents Not Used in the CBB Cycle May Be Effectively Exported From Chloroplasts Under ${\mathrm{NH}}_{4}^{+}$ Nutrition

The photosynthetic electron transport chain is not tightly linked to the CBB cycle because many different chloroplast-localized metabolic pathways, including nitrogen assimilation, sulfur assimilation, chloroplast redoxin functioning, etc., with different requirements for ATP and reductants, compete with one another. ${\mathrm{NH}}_{4}^{+}$ nutrition is one of the events, which are suspected to influence chloroplast redox and energy status. In fact in our previous study, we have shown that the status of adenylates in the chloroplasts was affected, and the plants were characterized by lower ATP/ADP ratio under ${\mathrm{NH}}_{4}^{+}$ nutrition (Podgórska et al., 2013). The observed ATP deficit in the ${\mathrm{NH}}_{4}^{+}$-nourished plants correlates with the lower ATP synthase complex abundance and the α-subunit of ATP synthase protein level (**Figures 3A** and **5A**), suggesting that less chloroplastic ATP may be produced during ${\mathrm{NH}}_{4}^{+}$ nutrition. Nonetheless, the elevated CET in the ${\mathrm{NH}}_{4}^{+}$-nourished plants could still contribute to a possibly increased ATP production. Certainly, our findings imply that lower ATP availability during ${\mathrm{NH}}_{4}^{+}$ nutrition affects the CO_2_ assimilation in CBB cycle (**Figure 5B**). A reduced CO_2_ assimilation rate was observed in ${\mathrm{NH}}_{4}^{+}$-grown rice (Alencar et al., 2019) and *Arabidopsis* seedlings (Hoffmann et al., 2007). Moreover, through their Northern blotting analysis, a slightly lower RUBISCO expression in *Arabidopsis* seedlings supplied with ${\mathrm{NH}}_{4}^{+}$ was further documented (Hoffmann et al., 2007). In our current study, ${\mathrm{NH}}_{4}^{+}$ nutrition lowered the abundance of RUBISCO in the plants (**Figure 5A**). It can be expected that, although the CBB cycle was restricted under ${\mathrm{NH}}_{4}^{+}$ nutrition (**Figures 5A, B**), the other energy and redox-consuming reactions were activated during ${\mathrm{NH}}_{4}^{+}$ nutrition. As previously anticipated by Podgórska et al. (2013), the higher activity of the GS-GOGAT cycle could alleviate the reductive stress in chloroplasts during ${\mathrm{NH}}_{4}^{+}$ nutrition.

Photorespiration is integral to stress response in green tissues (Voss et al., 2013). Adjustments in the photorespiratory cycle flow signify the need to export the excess reducing equivalents from chloroplasts, considering that the NADH obtained from the decarboxylation of Gly can be further oxidized in the mtETC. Therefore, photorespiratory reactions are deemed essential under ${\mathrm{NH}}_{4}^{+}$ nutrition (Guo et al., 2007). Contrary to that assumption and based on the protein abundance of photorespiratory enzymes GDC-H, SHMT and HPR (**Figure 7A**), we do not expect this process to be strongly activated in *Arabidopsis* during long-term ${\mathrm{NH}}_{4}^{+}$ nutrition. This subject still warrants further investigation, considering that elevated rates of photorespiration under ${\mathrm{NH}}_{4}^{+}$ nutrition were documented in *P. vulgaris* (Zhu et al., 2000) and in rice (Alencar et al., 2019). Another way for the efficient redox equivalent export from chloroplasts can be mediated by NADP^+^-MDH activity in the OAA/Mal shuttle (Scheibe, 2004). During ${\mathrm{NO}}_{3}^{-}$ nutrition, the lack of NADP^+^-MDH can be favorable to keep more reductants for reduction reactions (Selinski and Scheibe, 2014). However, our current findings imply that under certain circumstances such as ${\mathrm{NH}}_{4}^{+}$ nutrition and when excess reductants are available in the chloroplasts, a greater expression of *NADP* ^+^ *-MDH* (**Figure 7B**) together with the higher maximal activity of NADP^+^-MDH (**Figure 7C**) may facilitate reductant outflow towards the cytosol. In addition, an upregulated activity of NAD^+^-MDH isoforms under ${\mathrm{NH}}_{4}^{+}$ nutrition (**Supplementary Figure 8B**) may indicate that reductants are efficiently transferred between cellular compartments and are possibly oxidized in mitochondria, which are considered as cellular redox-balancing organelles.

### Reduced Expression of Mitochondrial AOX1a Modifies Photosynthetic Parameters Under NO_3_- Nutrition, But ${\mathrm{NH}}_{4}^{+}$ Does Not Increase Observed Changes

The major role of mitochondria is to regulate cellular redox balance (Noctor et al., 2007), considering the presence of alternative pathways in the mtETC (Noguchi and Yoshida, 2007; Rasmusson et al., 2008). Under intensive light conditions, AOX contributes more to prevent chloroplast/cellular redox poise (Yoshida et al., 2007). Similarly, our current findings indicate that the activity of AOX is crucial in plants nourished with ${\mathrm{NH}}_{4}^{+}$ when excess reductants need to be dissipated. In fact, we observed differences in the overexpressor plant growth rate under ${\mathrm{NH}}_{4}^{+}$ nutrition; the growth of XX-2 plants was less restricted (**Figure 9A**). Moreover, as a marker of oxidative damage to chloroplasts, the extent of lipid peroxidation of chloroplastic membranes was not induced in XX-2 when nourished with ${\mathrm{NH}}_{4}^{+}$ (**Supplementary Figure 3B**). Surprisingly, the modification of *AOX1a* expression did not drastically change the cellular metabolism of the plants under ${\mathrm{NH}}_{4}^{+}$ nutrition (Supplementary materials). Neither the overexpression nor suppression of *AOX1a* affected the H_2_O_2_ content of whole tissues in response to ${\mathrm{NH}}_{4}^{+}$ nutrition (**Figure 9B**). However, among the evaluated cellular ROS scavengers, which were not disturbed in the transformants (**Supplementary Figure 2**), only ascorbate was significantly affected. The antisense plants seemingly had to raise their total ascorbate content (**Figure 9C**) to achieve redox homeostasis. At the same time, the overexpressor mutants showed a more reduced ascorbate status when nourished with NH4+ (**Figure 9C**). Previous studies have shown that during optimal growth conditions, AOX1a-defective plants do not manifest alterations in their photosynthetic performance, or NPQ (Umbach et al., 2005; Giraud et al., 2008; Gandin et al., 2012; Vishwakarma et al., 2015), but the changes in the mtETC functioning of the AOX1a*-*suppressor plants may be a burden for them as they keep their photosynthetic rates efficient under stress. In the presence of stress factors such as intensive light and chilling or drought photoinhibition may intensify and ROS production may increase in AOX1a antisense plants (Watanabe et al., 2008; Giraud et al., 2008; Florez-Sarasa et al., 2011). Under ${\mathrm{NH}}_{4}^{+}$ nutrition, there were no parameters indicating photoinhibition of PSII because the F_V_/F_M_ values were similar among all the genotypes (**Figure 8A**). We have also not observed any influence of the overexpression of AOX1a on the photosynthetic parameters *in vivo* during light period (**Figure 8B**). Given that AOX is highly induced by ${\mathrm{NH}}_{4}^{+}$ nutrition (Escobar et al., 2006; Podgórska et al., 2018), we suppose that additional, genetically-forced increase in the quantity of AOX protein may have no cumulative effect on metabolic improvement, at least in the level of photosynthetic parameters. However, the dysfunction of AOX resulted in significant changes in the parameters characterizing photochemical quenching and the initial phase of NPQ (**Figure 8B**). Under the control ${\mathrm{NO}}_{3}^{-}$ conditions, the lower levels of AOX1a reduced the effective utilization of light-derived energy in photochemical reactions, but this effect was not modulated by nourishing the plants with reduced form of nitrogen. Contrary to the findings on the WT plants nourished with ${\mathrm{NH}}_{4}^{+}$, AS-12 plants did not show faster induction of NPQ in the first phase of light conditions. ${\mathrm{NH}}_{4}^{+}$ nutrition does not further alter photosynthetic performance in *AOX1a* knockout mutants (Hachiya et al., 2010; Gandin et al., 2014), similar to the findings in our current study.

## Data Availability Statement

All datasets generated and analyzed for this study are included in the article/**Supplementary Material**.

## Author Contributions

AP and BS designed the experiments. AP, MO-B, KB, and KK measured enzyme activity and performed Licor analysis, whereas AP and BS evaluated the metabolite levels. AP, KK, and KD conducted the RTq-PCR analysis. AP and RM performed the BN-PAGE. RM carried out the pigment analysis and PAM-imaging. BS, MO-B, AT, and MB worked on the immunoblots. AT did the statistical analysis, and MO-B prepared the figures. AP and BS wrote the manuscript, which was later revised by RM and AR.

## Funding

The publication process was co-financed from the resources of the University of Warsaw. Analysis of MDH was supported by the intramural grant 501/86/0112600-35 (DSM) provided to KB by the Ministry of Science and Higher Education through the Faculty of Biology, University of Warsaw.

## Conflict of Interest

The authors declare that the research was conducted in the absence of any commercial or financial relationships that could be construed as a potential conflict of interest.
