# Supplementary material for: Efficient Photosynthetic Functioning of Arabidopsis thaliana Through Electron Dissipation in Chloroplasts and Electron Export to Mitochondria Under Ammonium Nutrition
Source: Front Plant Sci. 2020 Feb 26;11:103. doi: 10.3389/fpls.2020.00103 (PMC7054346; doi:10.3389/fpls.2020.00103)
Supplement: Supplementary file 1 [file DataSheet_1.pdf]

Supplementary Material

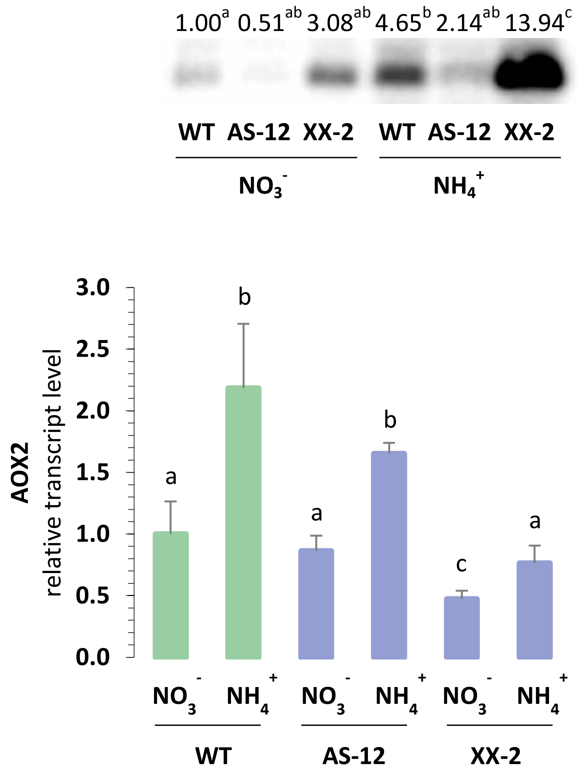

Supplementary Figure 1. Characterization of transgenic *AOX1a* (antisense, AS-12 or overexpression, XX-2) or WT plants grown with NH_4^+ or NO_3^- (control) as the only source of nitrogen. (A) Protein abundance of AOX1/2 (36-40 kDa). (B) Transcript level of AOX2. The relative expression was set to 1 in WT plants grown with NO_3^- (control) for reference. Representative immunoblot is shown out of n = 4 independent replicates. Bars or bands with different letters indicate significant difference ($p \leq 0.05$).

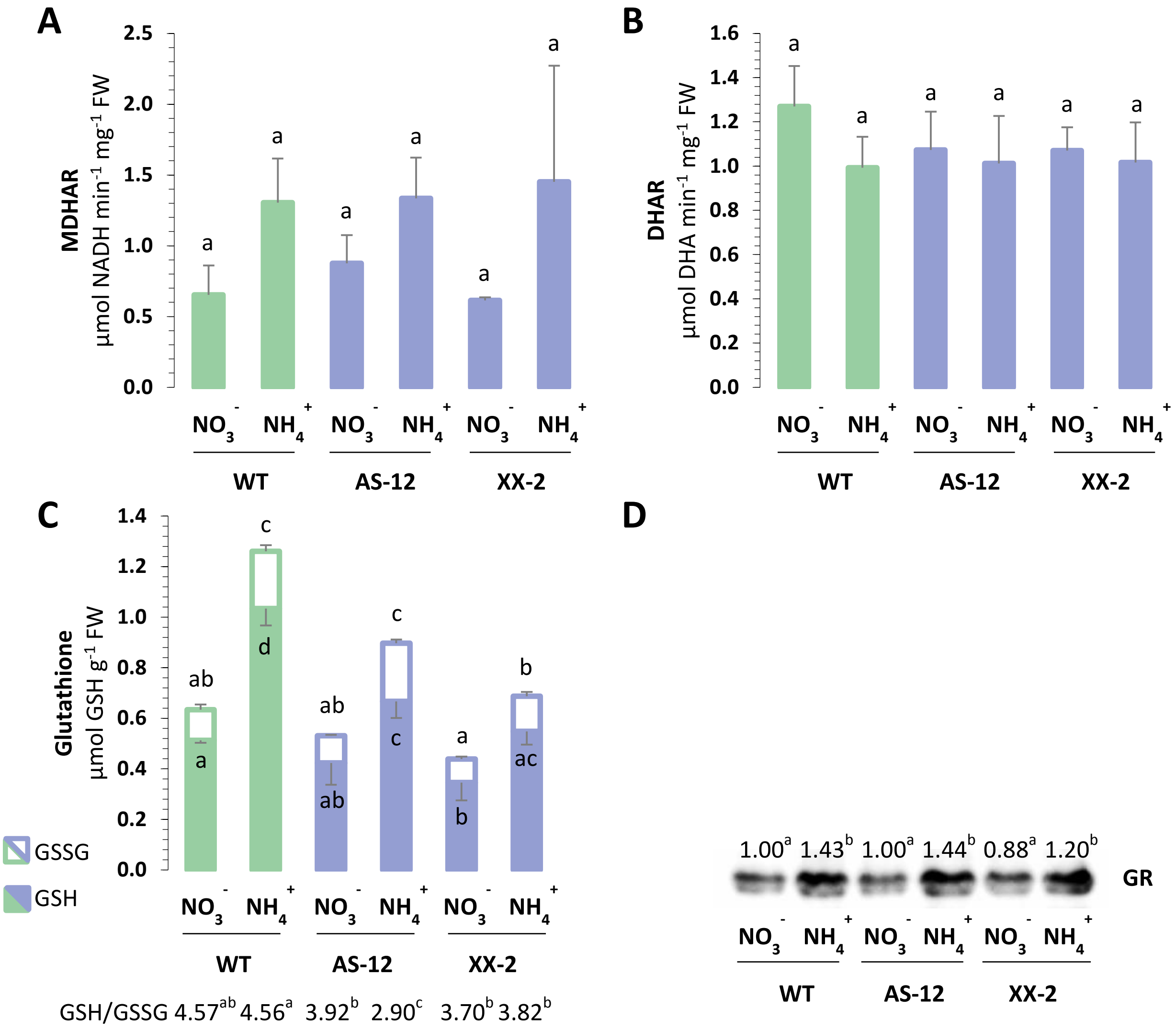

Supplementary Figure 2. Cellular antioxidant defense towards reactive oxygen species (ROS) in transgenic *AOX1a* (antisense, AS-12 or overexpression, XX-2) or WT plants grown with NH_4^+ or NO_3^- (control) as the only source of nitrogen. The activity of scavenger enzymes (A) monodehydroascorbate reductase (MDHAR) and (B) dehydroascorbate reductase (DHAR). (C) Concentration of glutathione. (D) Protein abundance of glutathione reductase (GR, 54 kDa). Representative immunoblot is shown out of n = 3–4 independent replicates. Bars or bands with different letters indicate significant difference ($p \leq 0.05$).

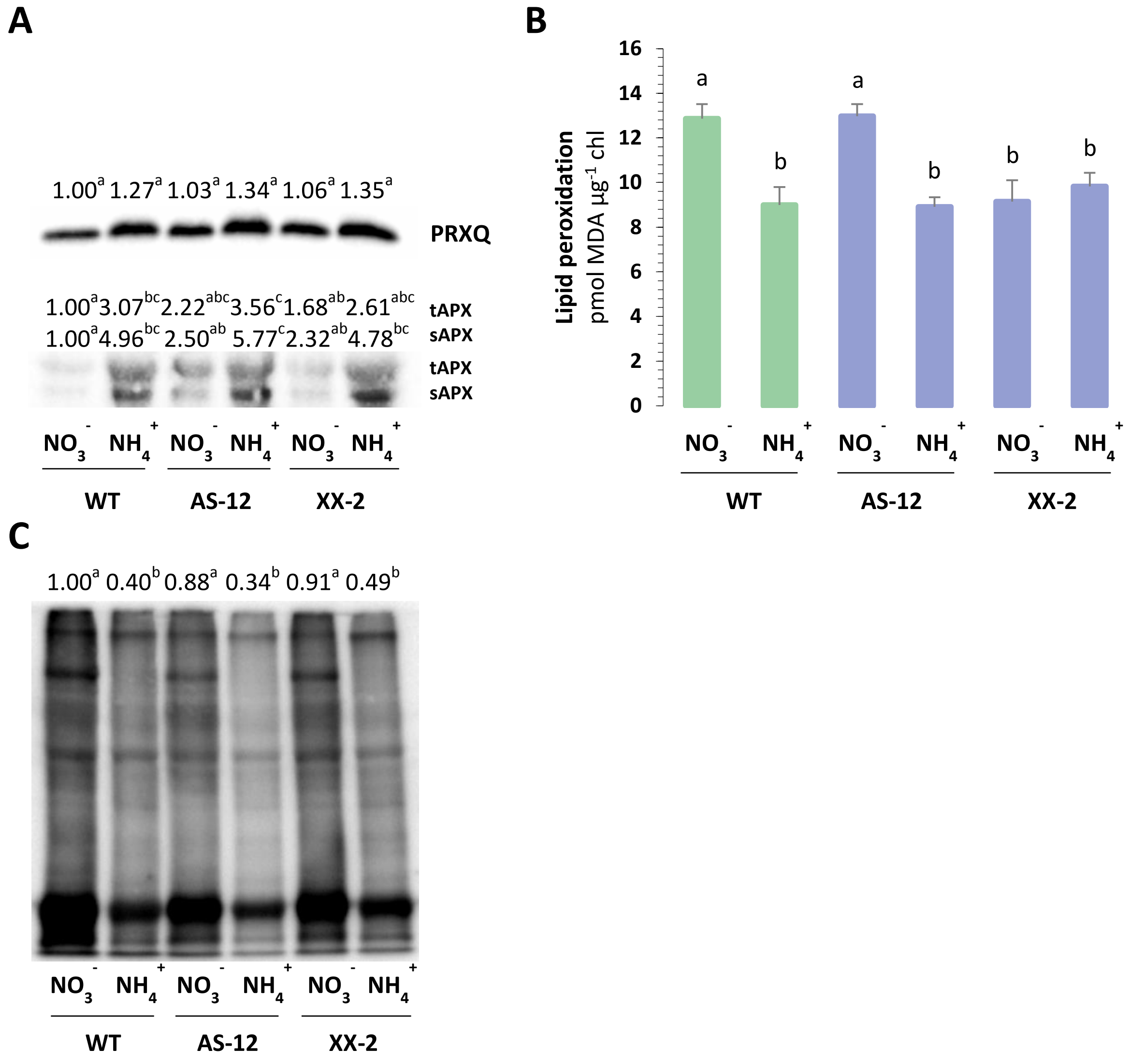

Supplementary Figure 3. Chloroplastic defense towards ROS and oxidative stress markers in transgenic *AOX1a* (antisense, AS-12 or overexpression, XX-2) or WT plants grown with NH_4^+ or NO_3^- (control) as the only source of nitrogen. (A) Protein abundance of chloroplast peroxiredoxin Q (PRXQ, 16 kDa) and thylakoid and stromal ascorbate peroxidase (tAPX, 38 kDa; sAPX, 33 kDa). (B) Lipid peroxidation in isolated chloroplasts. (C) Content of protein-bound carbonyls in chloroplasts. Representative immunoblots are shown out of n = 3–4 independent replicates. Bars or bands with different letters indicate significant difference ($p \leq 0.05$).

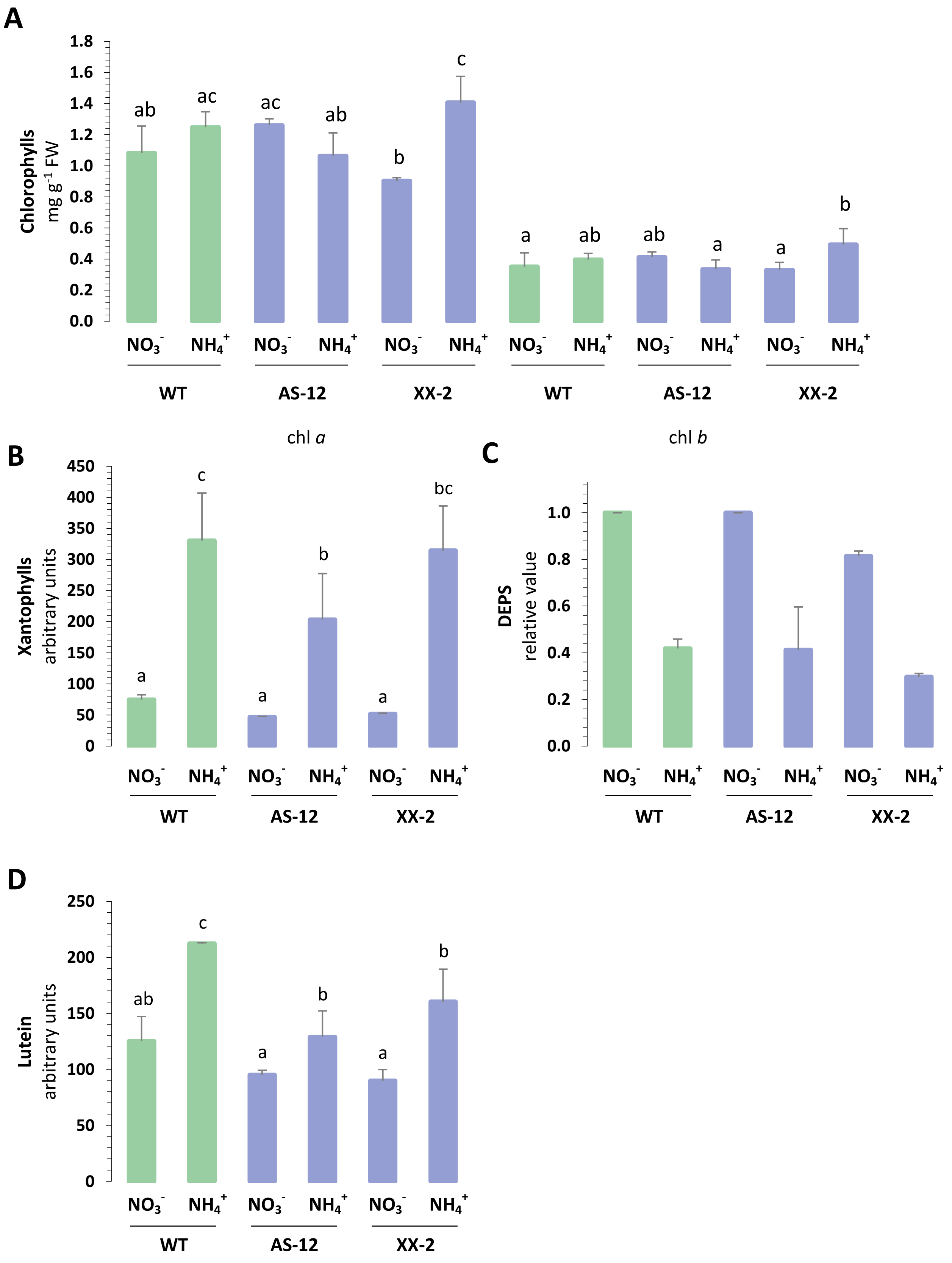

Supplementary Figure 4. Foliar pigment levels in transgenic *AOX1a* (antisense, AS-12 or overexpression, XX-2) or WT plants grown with NH_4^+ or NO_3^- (control) as the only source of nitrogen. Levels of (A) chlorophyll (chl) *a*, and chl *b* and (B) xanthophyll cycle pigments. (C) De-epoxidation state (DEPS) of xanthophylls. (D) Levels of lutein. Bars with different letters indicate significant difference ($p \leq 0.05$).

A

	WT		AS-12		XX-2	
	NO_3^-	NH_4^+	NO_3^-	NH_4^+	NO_3^-	NH_4^+
PSI-NDH	1.00[a]	1.41[b]	1.01[a]	1.27[b]	1.05[a]	1.37[b]
PSII-LHCII	1.00[a]	1.56[b]	1.16[a]	1.57[b]	1.21[a]	1.71[b]
ATPase	1.00[a]	0.78[b]	0.96[a]	0.81[b]	0.92[a]	0.83[b]

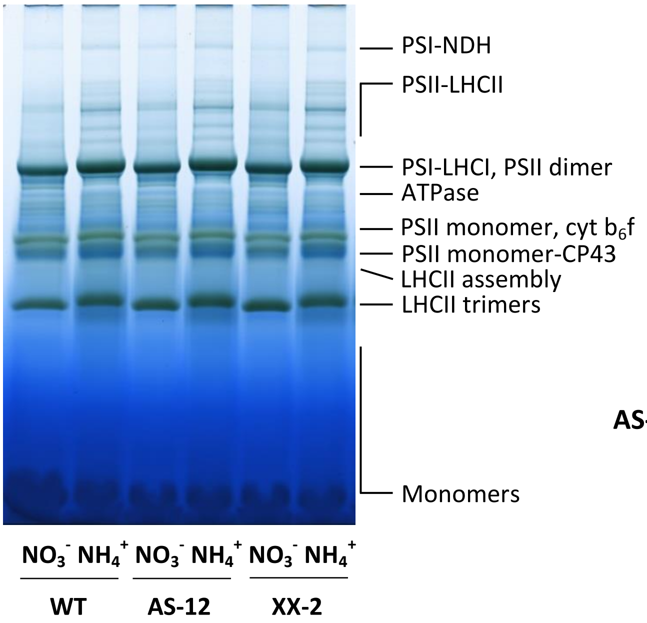

B

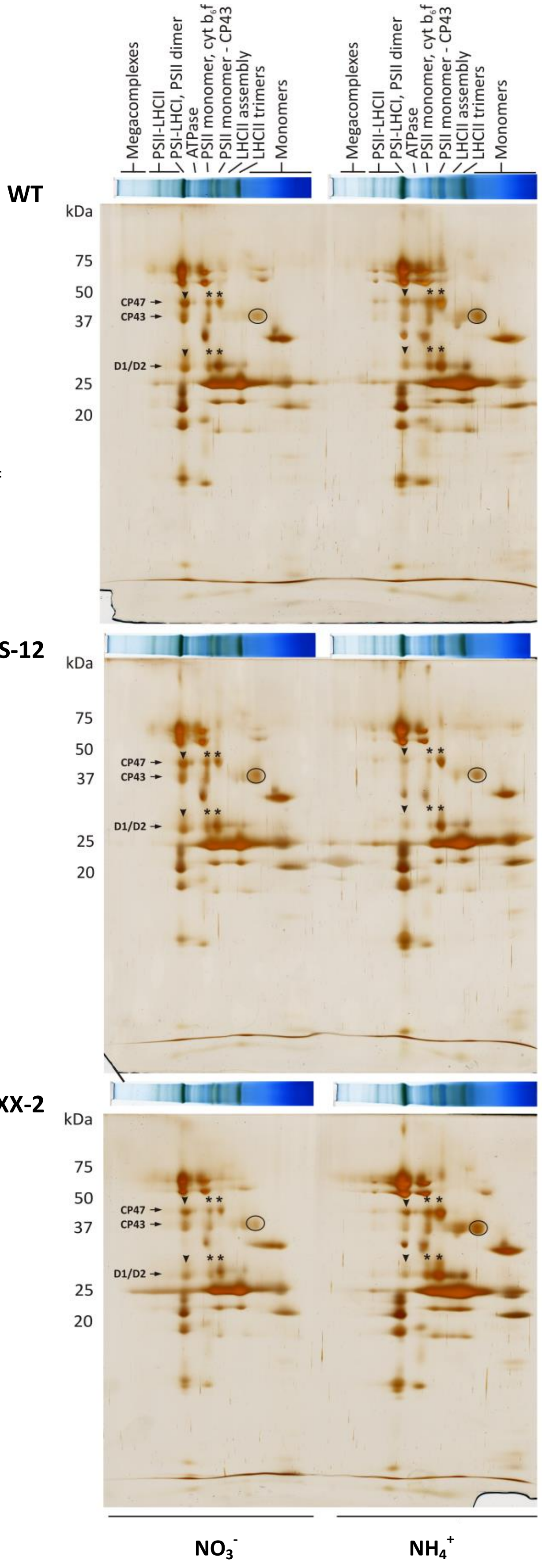

Supplementary Figure 5. Composition of the photosynthetic protein complexes in transgenic *AOX1a* (antisense, AS-12 or overexpression, XX-2) or WT plants grown with NH_4^+ or NO_3^- (control) as the only source of nitrogen. (A) Identification of thylakoid protein complexes in blue native - polyacrylamide gel electrophoresis (BN-PAGE) gel. Thylakoid membranes were solubilized in 1% dodecyl maltoside (DDM), and the protein extracts containing 8.3 µg of chlorophyll were separated by electrophoresis. Bands with different letters quantified by densitometry indicate significant difference ($p \leq 0.05$). (B) Patterns of thylakoid protein complexes in second dimension by 2D-PAGE. BN-PAGE strips were denatured and separated on sodium dodecyl sulfate (SDS)-Urea-PAGE and were silver-stained. Representative gels are shown out of n = 3–6 independent replicates.

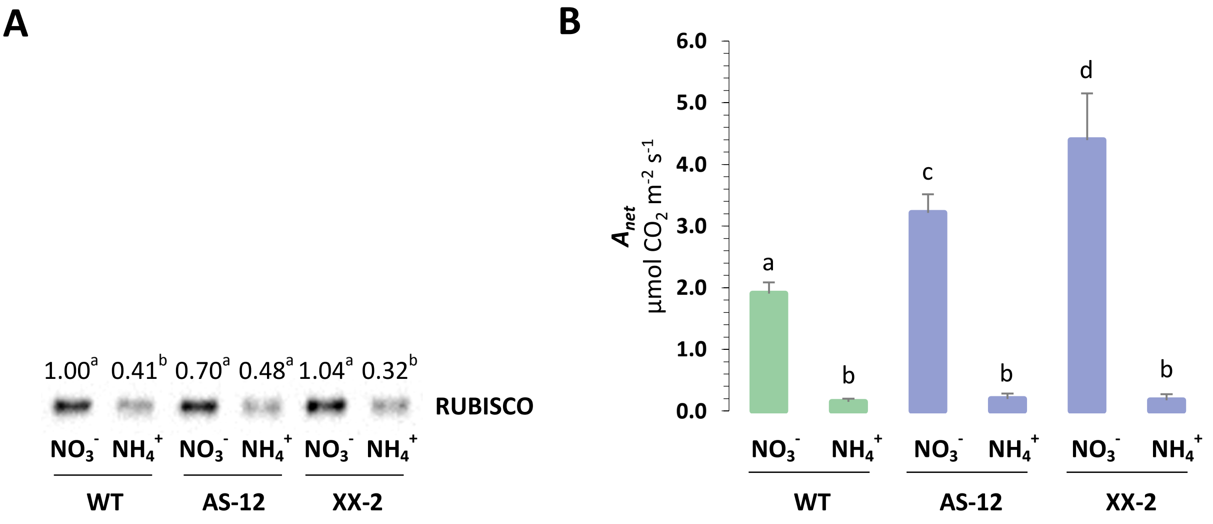

Supplementary Figure 6. Photosynthetic performance in transgenic *AOX1a* (antisense, AS-12 or overexpression, XX-2) or WT plants grown with NH_4^+ or NO_3^- (control) as the only source of nitrogen. (A) Ribulose-1,5-bisphosphate-carboxylase/oxygenase protein abundance (RUBISCO, 50 kDa). (B) Photosynthesis net rate (A_{net}). Representative immunoblots are shown out of n = 3–4 independent replicates. Bars or bands with different letters indicate significant difference ($p \leq 0.05$).

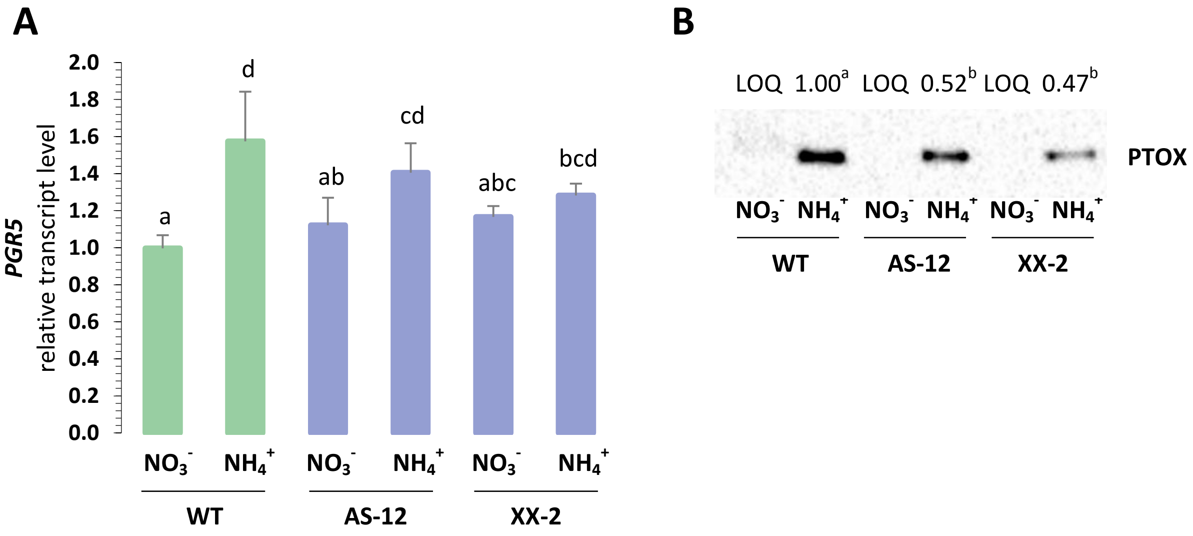

Supplementary Figure 7. Electron dissipation from the chloroplast electron transport chain in transgenic *AOX1a* (antisense, AS-12 or overexpression, XX-2) or WT plants grown with NH_4^+ or NO_3^- (control) as the only source of nitrogen. (A) Proton gradient regulation 5 (*PGR5*) transcript level. The relative expression was set to 1 in WT plants grown with NO_3^- (control) for reference. (B) Plastid terminal oxidase protein abundance (PTOX, 37 kDa). Representative immunoblot is shown out of n = 4 independent replicates. Bars or bands with different letters indicate significant difference ($p \leq 0.05$). LOQ, under limit of quantification.

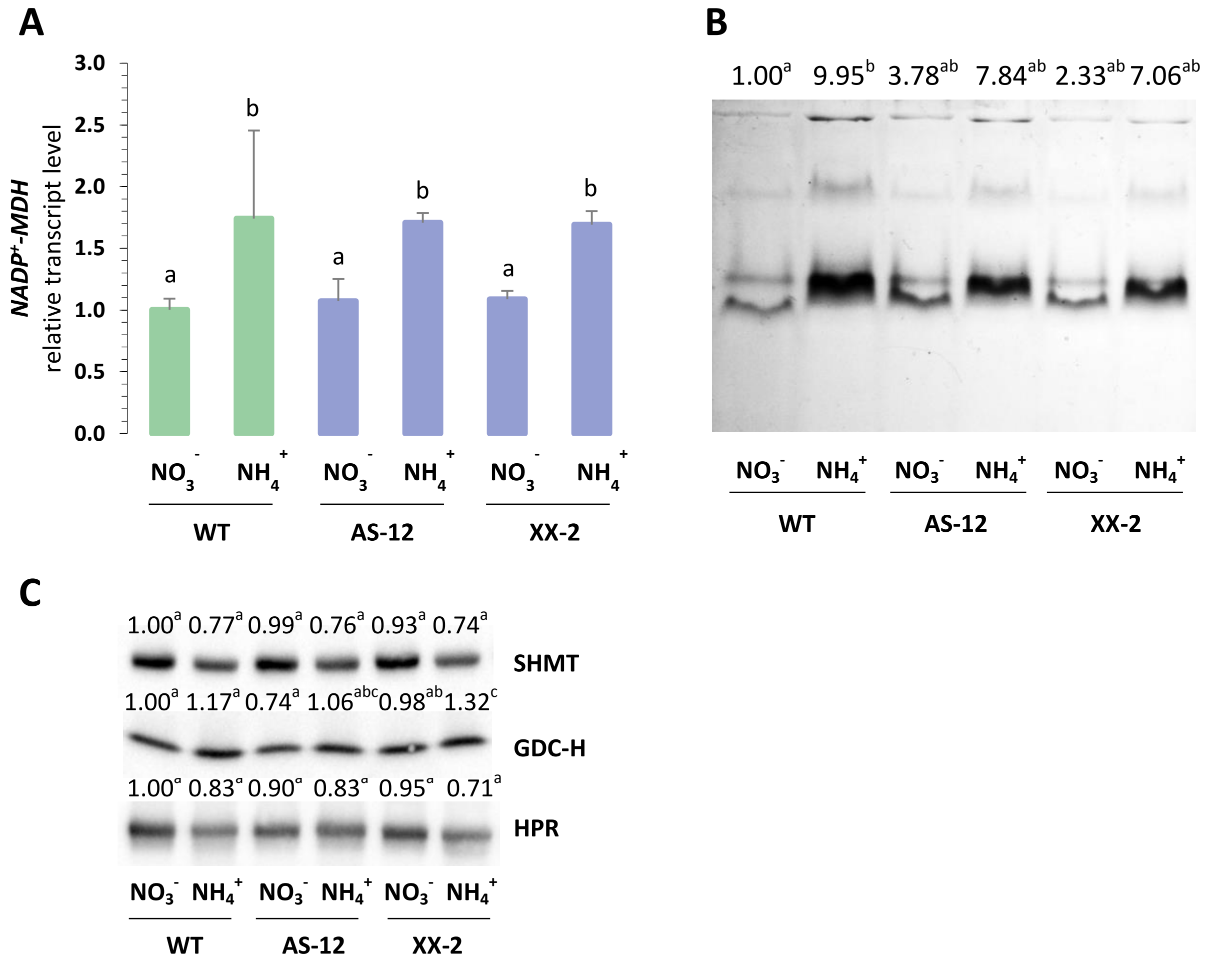

Supplementary Figure 8. Export of reductants out of chloroplasts in transgenic *AOX1a* (antisense, AS-12 or overexpression, XX-2) or WT plants grown with NH_4^+ or NO_3^- (control) as the only source of nitrogen. (A) Transcript level of chloroplast $NADP^+$-malate dehydrogenase (*$NADP^+$-MDH*). The relative expression is set to 1 in WT plants grown with NO_3^- (control) for reference. (B) In-gel activity of NAD^+-MDH. (C) Protein abundance of mitochondrial serine hydroxymethyltransferase (SHMT, 50 kDa), mitochondrial glycine decarboxylase complex H subunit (GDC-H, 16 kDa), and peroxisome hydroxypyruvate reductase (HPR, 43 kDa). Representative gel and immunoblots are shown out of n = 3–4 independent replicates. Bars or bands with different letters indicate significant difference ($p \leq 0.05$).

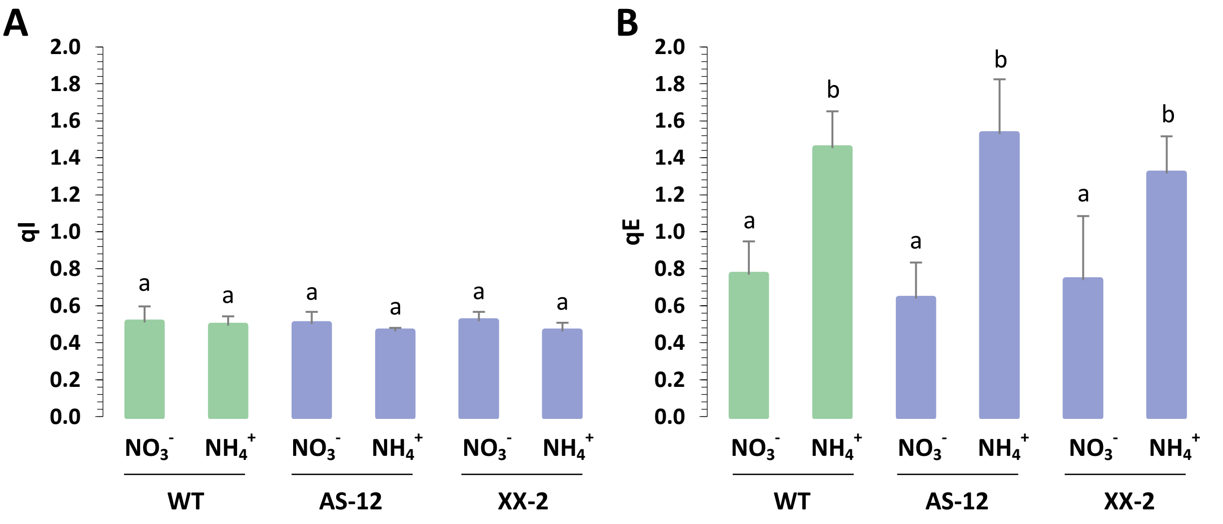

Supplementary Figure 9. The efficiency of photosynthetic light reactions in transgenic *AOX1a* (antisense, AS-12 or overexpression, XX-2) or WT plants grown with NH_4^+ or NO_3^- (control) as the only source of nitrogen. Non-photochemical quenching (NPQ) component: (A) qI; and (B) qE. Data are mean values ± SD from at least eight leaves. Results with different letters indicate significant difference ($p \leq 0.05$).
